# NEDD4 family ubiquitin ligases associate with LCMV Z’s PPXY domain and are required for virus budding, but not via direct ubiquitination of Z

**DOI:** 10.1371/journal.ppat.1008100

**Published:** 2019-11-11

**Authors:** Christopher M. Ziegler, Loan Dang, Philip Eisenhauer, Jamie A. Kelly, Benjamin R. King, Joseph P. Klaus, Inessa Manuelyan, Ethan B. Mattice, David J. Shirley, Marion E. Weir, Emily A. Bruce, Bryan A. Ballif, Jason Botten

**Affiliations:** 1 Department of Medicine, Division of Immunobiology, University of Vermont, Burlington, Vermont, United States of America; 2 Cellular, Molecular and Biomedical Sciences Graduate Program, University of Vermont, Burlington, Vermont, United States of America; 3 Ixis LLC, Data Science Division, Burlington, Vermont, United States of America; 4 Department of Biology, University of Vermont, Burlington, Vermont, United States of America; 5 Department of Microbiology and Molecular Genetics, University of Vermont, Burlington, Vermont, United States of America; The Scripps Research Institute, UNITED STATES

## Abstract

Viral late domains are used by many viruses to recruit the cellular endosomal sorting complex required for transport (ESCRT) to mediate membrane scission during viral budding. Unlike the P(S/T)AP and YPX_(1–3)_L late domains, which interact directly with the ESCRT proteins Tsg101 and ALIX, the molecular linkage connecting the PPXY late domain to ESCRT proteins is unclear. The mammarenavirus lymphocytic choriomeningitis virus (LCMV) matrix protein, Z, contains only one late domain, PPXY. We previously found that this domain in LCMV Z, as well as the ESCRT pathway, are required for the release of defective interfering (DI) particles but not infectious virus. To better understand the molecular mechanism of ESCRT recruitment by the PPXY late domain, affinity purification-mass spectrometry was used to identify host proteins that interact with the Z proteins of the Old World mammarenaviruses LCMV and Lassa virus. Several Nedd4 family E3 ubiquitin ligases interact with these matrix proteins and in the case of LCMV Z, the interaction was PPXY-dependent. We demonstrated that these ligases directly ubiquitinate LCMV Z and mapped the specific lysine residues modified. A recombinant LCMV containing a Z that cannot be ubiquitinated maintained its ability to produce both infectious virus and DI particles, suggesting that direct ubiquitination of LCMV Z alone is insufficient for recruiting ESCRT proteins to mediate virus release. However, Nedd4 ligases appear to be important for DI particle release suggesting that ubiquitination of targets other than the Z protein itself is required for efficient viral ESCRT recruitment.

## Author summary

Enveloped viruses derive their lipid bilayer from either the cellular plasma membrane or an intracellular organelle during the process of viral budding in which a virus particle is formed at a membrane. Many enveloped viruses recruit the cellular endosomal sorting complex required for transport (ESCRT) in order to efficiently cut the membrane that connects a newly budded, but not released, virus particle from its parent membrane. Late domains, which are short protein motifs found in numerous enveloped viruses, specifically recruit ESCRT for this process. Two types of late domains accomplish this by binding directly to ESCRT proteins. A third late domain, PPXY, recruits ESCRT proteins through an unknown, indirect linkage. In this study, we sought to identify proteins that may bridge the PPXY late domain and ESCRT proteins. We found that Nedd4 family ubiquitin ligases interact with the PPXY domain in the mammarenavirus Z protein resulting in ubiquitination of Z at two lysine residues. However, Z ubiquitination was largely dispensable for the virus. Conversely, Nedd4 ubiquitin ligases were critical during infection suggesting that the most important contribution made to virus release by Nedd4 ligases is not direct ubiquitination of the viral matrix protein, but possibly the ubiquitination of cellular proteins or other viral proteins.

## Introduction

The mammalian endosomal sorting complex required for transport (ESCRT) mediates scission of membrane stalks formed when membrane-bound vesicles bud from their parent membranes in a direction away from the cytoplasm [1, 2]. These reverse topology membrane biogenesis events are distinct from membrane budding events toward the cytoplasm, such as endocytosis, and use entirely different protein complexes to accomplish membrane scission [1]. The ESCRT pathway functions in a variety of endogenous cellular processes including multivesicular body formation [3–5], shedding of microvesicles [6], exosome formation [7, 8], micro- and macro-autophagy [9–11], cytokinesis [12, 13] and an expanding list of other cellular processes [8]. ESCRT consists of four protein complexes (ESCRT-0, -I, -II and -III) and the accessory proteins ALIX, VPS4A/B, IST1 and Spastin [14]. Function of this pathway requires the assembly of a stable scaffold formed by ESCRT-0, I and/or II which are recruited to sites of membrane budding and subsequently recruit the ESCRT-III complex that mediates membrane scission [1, 15]. Finally, disassembly of the complex is mediated by the AAA-ATPase VPS4A/B or Spastin [1]. ALIX (also known as PDCD6IP) can recruit ESCRT-III directly or substitute for ESCRT-0 by binding to the ESCRT-I complex [16, 17]. Recruitment of ESCRT to sites of membrane biogenesis is initiated by adaptor proteins that bind to ESCRT components. The cellular proteins ARRDC1, CEP55, and Syntenin serve as adaptor proteins for microvesicle shedding [6], cellular abscission [12] and exosome formation [18], respectively. A ubiquitin modification can also serve as an entry point to the ESCRT pathway as exemplified by ubiquitinated endosomal cargo proteins that bind the ubiquitin binding domains in the ESCRT-0 complex [19–21].

A wide range of viruses also use the ESCRT scission machinery to facilitate release of new virions where viral proteins serve as adaptors for recruiting ESCRT proteins [2]. These viral adaptor proteins encode short peptides called late domains that mediate the recruitment of ESCRT complexes. To date there are four classes of late domains identified: P(S/T)AP, originally found in the human immunodeficiency virus 1 Gag protein [22, 23]; YPX_(1,3)_L, originally discovered in the Gag protein of equine infectious anemia virus Gag [24]; PPXY, found first in the Rous sarcoma virus Gag protein [25]; and FPIV, found in paramyxoviruses [26, 27]. The P(S/T)AP, YPX_(1,3)_L, and PPXY late domains have since been discovered in numerous other viruses and, importantly, have been identified in several key cellular proteins [2]. A P(S/T)AP motif is found in the ESCRT-0 protein Hrs as well as in the microvesicle adaptor protein ARRDC1 [6, 19]. YPX_(1,3)_L is present in the exosome adaptor protein syntenin [18]. Late domains function by facilitating protein-protein interactions between cellular or viral adaptor proteins and ESCRT proteins. Specifically, the P(S/T)AP late domain can directly bind the ESCRT-I protein, Tsg101 [28–30], while the YPX_(1,3)_L late domain directly binds to the ESCRT accessory protein, ALIX [31]. ALIX, in turn, is capable of binding to both the ESCRT-III complex or to Tsg101 through its own P(S/T)AP motif [16, 17, 32, 33]. While those molecular interactions are well-defined, the link between FPIV and PPXY late domains and the ESCRT complexes is unclear [2].

A diverse range of viruses encode PPXY late domains to mediate virus release including several retroviruses, rhabdoviruses, filoviruses, Old World mammarenaviruses, hepatitis B virus and Bluetongue virus (reviewed in [2]). Unlike the P(S/T)AP and YPX_(1,3)_L late domains, the PPXY motif does not appear to directly bind to ESCRT proteins. PPXY domains, however, can directly bind to the WW domains found in Nedd4 family E3 ubiquitin ligases, which are not part of the ESCRT pathway [34–37]. Several lines of evidence suggest that PPXY-dependent viral budding requires ubiquitination of cellular or viral proteins by this family of ligases. First, interaction with these ligases can result in ubiquitination of the PPXY-containing protein as has been shown for filovirus VP40 [38, 39], retroviral Gag [40–43], and the vesicular stomatitis M protein [44]. Second, depletion of the intracellular ubiquitin pool decreases budding activity for several viruses [41, 44–46] and viral late domains can be functionally substituted for by artificial conjugation of ubiquitin to the viral matrix protein [45, 47, 48]. Furthermore, several early-acting ESCRT proteins contain ubiquitin binding domains [49]. Notably, the ubiquitin ligase activity of Nedd4 family proteins is required for efficient virus particle egress for filoviruses [38, 50, 51], human T-cell leukemia virus [40, 52], murine leukemia virus [53, 54] and Rous Sarcoma virus [42].

Viruses rely on components of their cellular hosts in order to complete their life cycle. Identification of the cellular proteins hijacked by viral proteins to accomplish their various functions is critical for enhancing our understanding of basic viral processes. Recent studies in the mammarenavirus field have revealed some of these critical interactions using a proteomics approach [55–60]. Mammarenaviruses are a genus within the arenaviridae family [61, 62]. They are zoonotic viruses carried primarily by rodents that can be transmitted to humans and cause disease ranging in severity from mild infections to severe hemorrhagic fever [63, 64]. Lassa virus is a major cause of mammarenavirus hemorrhagic fever and significant outbreaks have occurred in western Africa recently [65–68]. A closely related mammarenavirus, lymphocytic choriomeningitis virus (LCMV), can cause a mild, flu-like infection or more severe aseptic meningitis in healthy adults [69], but is also of significant concern during pregnancy and in solid organ transplantation [69–73]. These viruses have simple genomes that encode only four proteins, including the Z protein, which is a viral matrix protein that drives release of virus particles [74, 75]. Both LCMV and LASV Z proteins encode a PPXY late domain, while LASV encodes an additional P(S/T)AP late domain [74, 75]. Our lab has recently shown that the PPXY late domain in LCMV Z as well as the ESCRT pathway are required for efficient formation of defective interfering particles but are dispensable for infectious virus release [76]. Given the limited understanding of how PPXY late domains recruit the ESCRT pathway for any virus family, we sought to identify cellular proteins that interact with the LCMV and LASV Z proteins in order to gain a better understanding of this process. Mass spectrometry was used to identify interactions between these two Z proteins and their cellular partners. Among a diverse set of proteins, four Nedd4 family ubiquitin ligases were identified as partners of Z. These ligases ubiquitinate LCMV Z at two lysine residues and loss of these ubiquitination sites in Z did not significantly alter the amount of virus particles released. However, loss of Nedd4 family ligases inhibited release of LCMV DI particles. Thus, this study rules out direct ubiquitination of LCMV Z as a primary mechanism for PPXY-mediated recruitment of ESCRT for viral budding and suggests that ubiquitination of other Z-associated protein partners may drive this process.

## Results

### Mapping the protein interactome of LCMV Z, LASV Z, and intact LCMV virions

**Fig 1:**
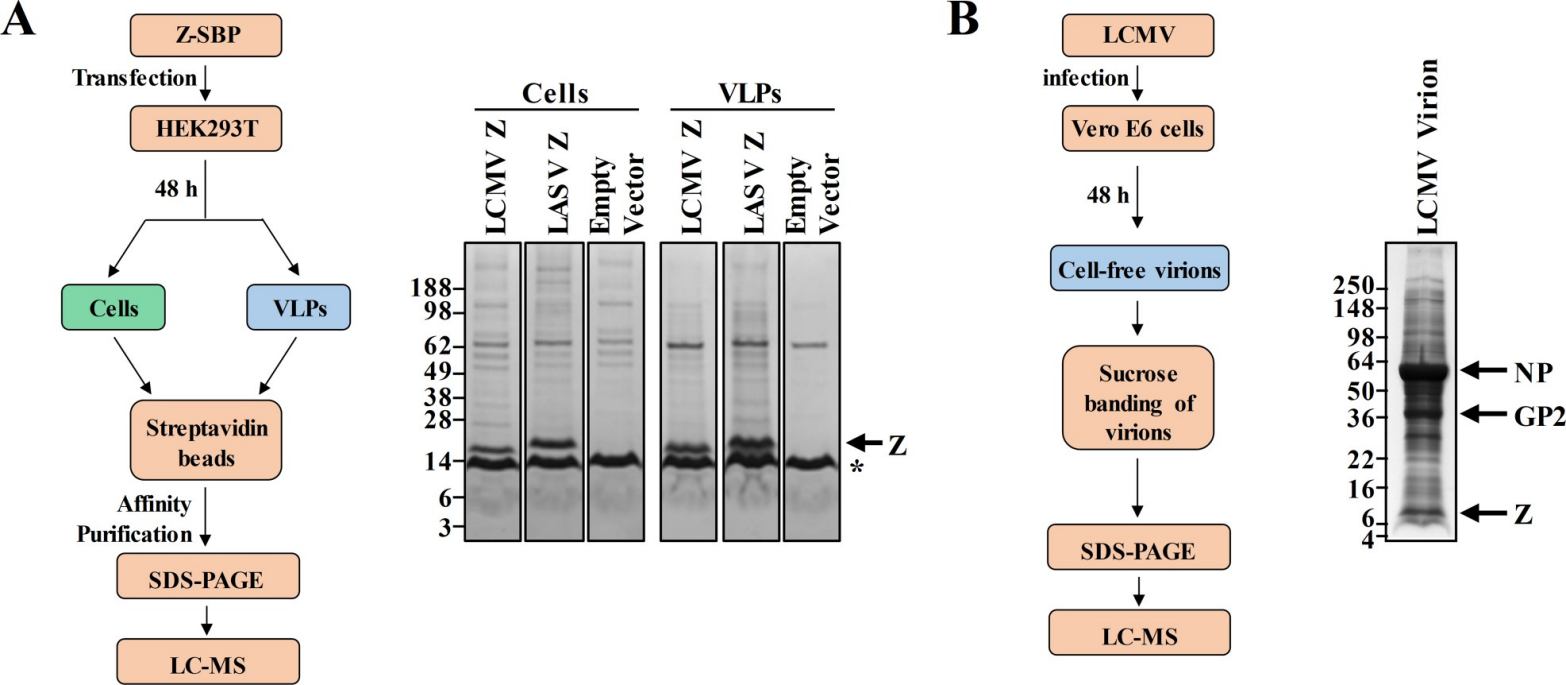
Identification of host proteins that interact with the LCMV and LASV Z matrix proteins or are packaged into LCMV virions. (A) The flow diagram depicts the affinity purification- and mass spectrometry-based approach used to identify human protein partners of LCMV or LASV Z proteins. HEK293T cells were transfected with a plasmid encoding LCMV or LASV Z with a C-terminal streptavidin binding peptide (SBP) tag or an empty vector. Z and its human protein binding partners were affinity purified from cell lysates or cell-free virus-like particles (VLPs) using magnetic streptavidin beads and the captured protein complexes were separated by protein gel electrophoresis and processed for mass spectrometry as described in the Methods. The Coomassie-stained protein gel shown is representative of two independent experiments. (B) The flow diagram shows the sucrose banding- and mass spectrometry-based approach to detect host proteins in LCMV particles. Intact virions of LCMV strain Armstrong 53b produced in Vero E6 cells were sucrose-banded and then subjected to lysis followed by protein gel electrophoresis. The Coomassie stained gel, which was processed for mass spectrometry as described in the Methods, is shown and various viral proteins are indicated.

To uncover the interactome of the LCMV and LASV Z proteins we conducted several experiments summarized in Fig 1A and S1 Table. From two independent experiments, a total of 744 cellular proteins were identified as partners of LCMV Z and 824 proteins for LASV Z in at least one independent experiment in cells, VLPs, or both (S1 Table). A total of 615 host proteins were identified in LCMV VLPs and 305 co-purified with intracellular LCMV Z from two independent experiments, while 27.3% and 43.6% of these proteins, respectively, were identified in both independent experiments. For LASV Z, 616 and 411 total proteins were identified in VLPs or interacting with intracellular Z in two independent experiments, respectively, of which 181 and 192 proteins were identified in both independent experiments (S1 Table). Of note, the overall sensitivity of detection for the second independent experiment appeared to be significantly greater than the first as there were nearly three-fold more total peptides identified in this second replicate experiment (S1 Table). To complement the identification of proteins found in LCMV VLPs, the cellular protein content of intact virions from LCMV strain Armstrong 53b was analyzed (Fig 1B). A total of 143 proteins were identified in virions of which 57 (39.9%) were also found in LCMV VLPs in both replicate experiments (S1 Table). An additional 24 of the virion-associated proteins were found in LCMV VLPs in only the second replicate experiment where the sensitivity of detection was greater (S1 Table). Notably, the virions were produced in Vero E6 cells, which are African green monkey cells, and the resulting peptides identified by the mass spectrometer were compared to a human protein database, limiting detection in cases of more divergent proteins. While not all of the proteins found in LCMV VLPs were detected in *bona fide* virions, several key proteins were found in both categories, including the ESCRT protein ALIX, also known by its official gene symbol, PDCD6IP (S1 Table).

**Fig 2:**
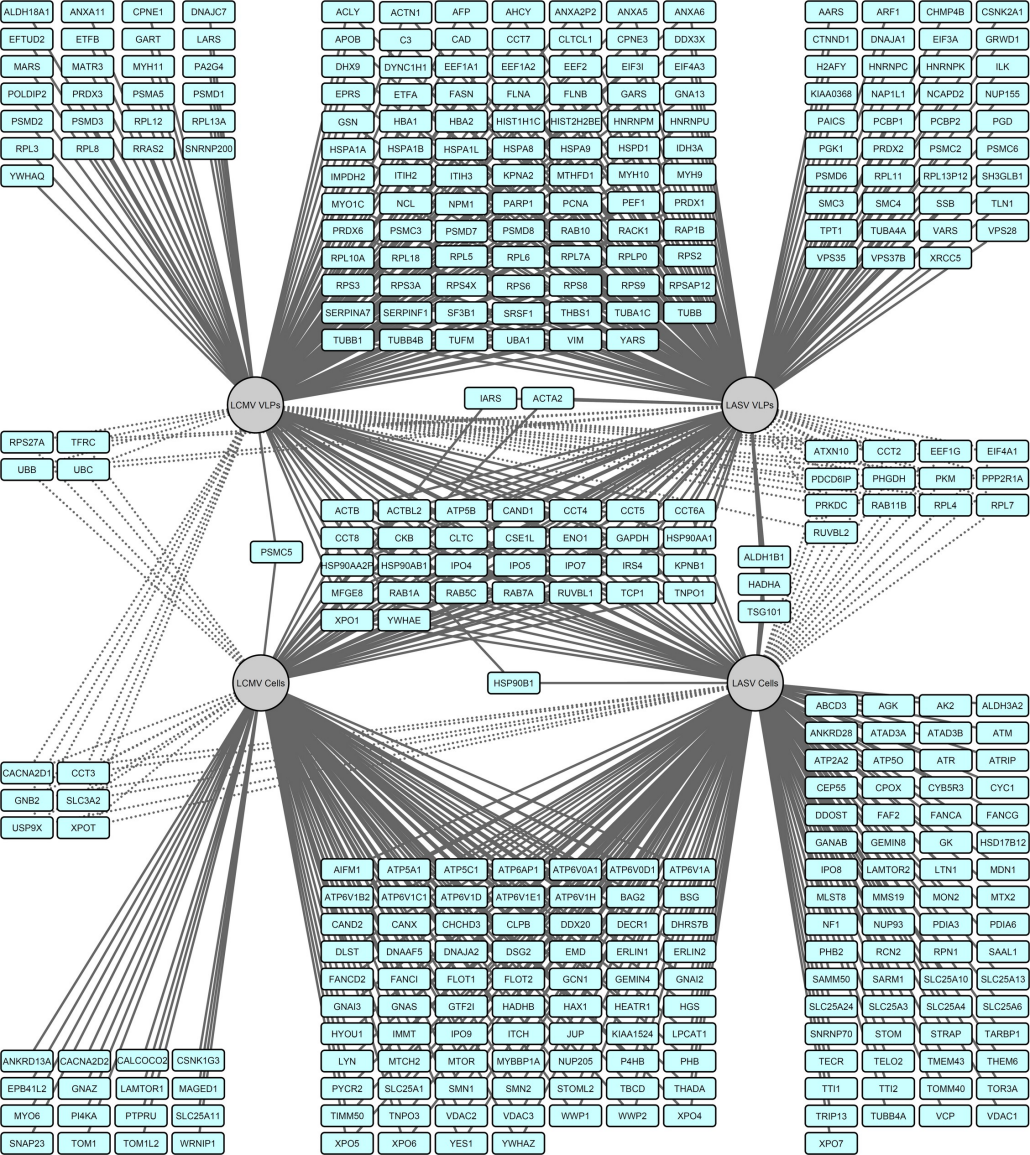
Old World Z-host protein interaction network. A network map of the protein-protein interactions between either LCMV or LASV Z and host proteins purified from cells or VLPs was generated using Cytoscape 3.2.1 software. Only proteins identified in both biological replicate experiments are depicted on the map. Solid lines indicate host proteins identified in 1, 2, or 4 conditions and dashed lines indicate host proteins identified in 3 conditions.

Fig 2 illustrates the proteins identified with either LCMV or LASV Z purified from cells or VLPs. A relatively small proportion of the total LCMV or LASV Z protein partners were identified in both the VLP and intracellular interactomes suggesting that the protein environments in those two contexts are somewhat distinct (Fig 2 and S1 Table). However, a significant number of protein interactions were conserved in the LCMV and LASV Z intracellular or extracellular interactomes (Fig 2 and S1 Table). These interactions that are conserved between the two virus species may represent cellular machinery and processes that are broadly critical for mammarenavirus infection. There were, however, a number of interactions exclusive to one virus species. For example, Tsg101 consistently co-purified with LASV Z but not LCMV Z (Fig 2 and S1 Table). Tsg101 is an ESCRT protein that binds to P(S/T)AP late domains, which LASV Z has but LCMV Z does not. Thus, determining how other cellular proteins that interact specifically with one species contribute to Z protein function and to the overall virus life cycle may provide clues to understanding the basis of biological differences between the two viruses.

### Bioinformatic analysis of Z interactome

**Fig 3:**
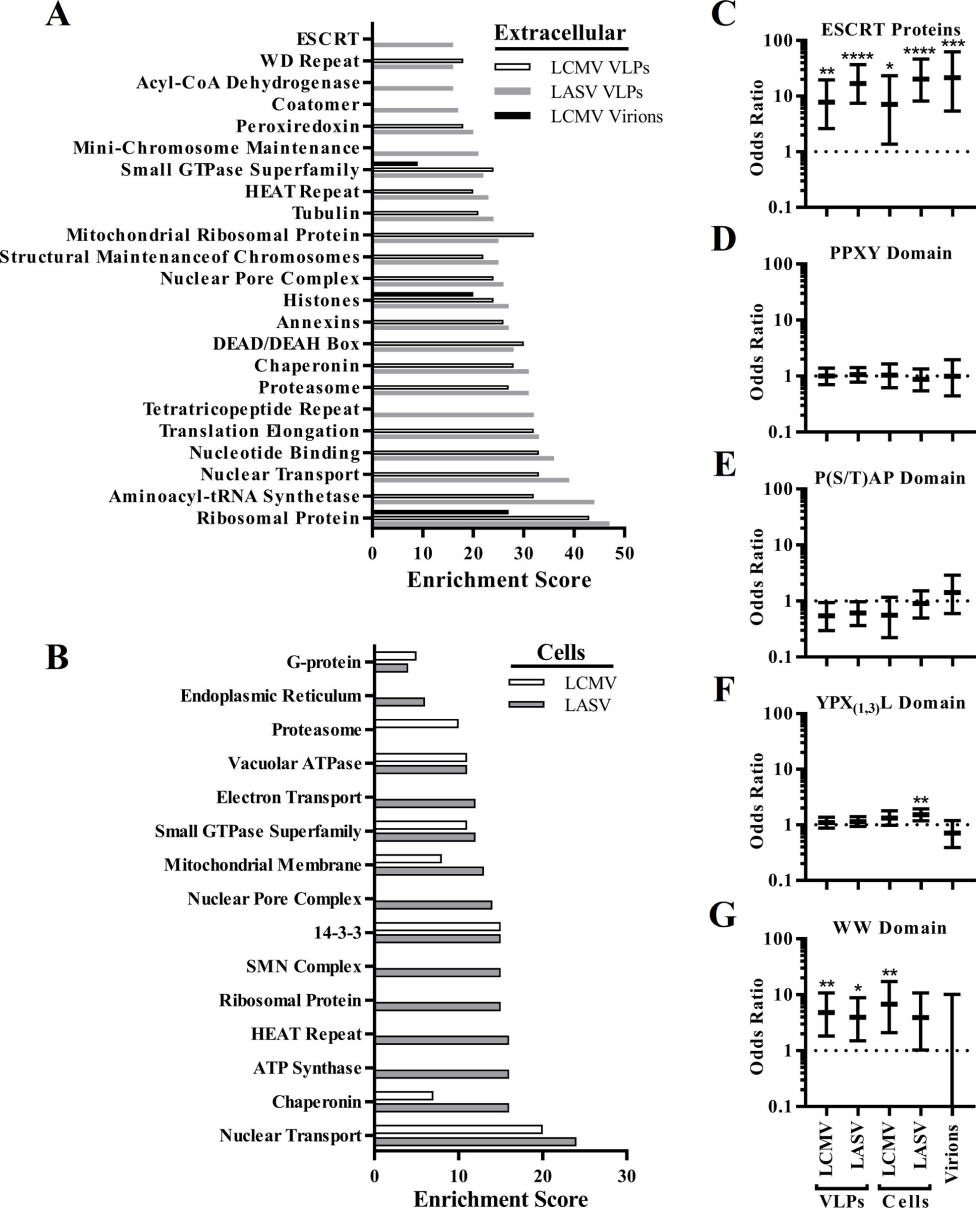
Bioinformatic analysis of the Z interactome. (A-B) The DAVID gene functional classification tool (version 6.8) with the high stringency setting was used to identify classes of proteins that were enriched in the LCMV or LASV Z VLP (A) or intracellular (B) interactomes. Proteins enriched in LCMV virions are also shown (A). (C-E) Overrepresentation test for manually curated classes of proteins. All human proteins that are part of the ESCRT pathway (C), contain PPXY (D), P(S/T)AP (E) or YPX(1,3)L (F) late domains, or contain WW domains (G) were identified and cross-referenced with the Z interactome and LCMV virion content. An odds ratio, confidence interval, and p-value were calculated for each in order to determine whether each protein class was enriched in the dataset where *p < 0.05, ** p < 0.01, *** p < 0.001, and ****p < 0.0001. (A-G) Proteins that were identified in at least one independent experiment were included in these analyses.

To characterize the proteins represented in the Z and LCMV virion interactomes, the DAVID Gene Functional Classification tool (version 6.8) [77, 78] was used to identify classes of proteins that were enriched among the different classes of Z binding partners or LCMV virions (Fig 3A and 3B and S2 Table). Ribosomal proteins were the most highly enriched proteins in VLPs and LCMV virions, which fits with early studies of mammarenavirus particles in which electron micrographs revealed electron-dense granules in virions that resembled host ribosomes and for which the virus family was named [79]. Host proteins containing specific structural domains were enriched in VLPs including the WD repeat, HEAT repeat, DEAD/DEAH box, and tetratricopeptide repeat (Fig 3A). Various proteins involved in intracellular vesicle transport were part of the Z interactome including small GTPase superfamily, coatomer complex, vacuolar ATPase, and ESCRT proteins (Fig 3A and 3B). Small GTPase superfamily proteins were also enriched in LCMV virions (Fig 3A). Interestingly, while Z has a predominately cytoplasmic localization, nuclear transport proteins and components of the nuclear pore complex were highly represented in the Z interactome (Fig 3A and 3B). Largely, the classes of proteins enriched in the LASV Z VLPs were also enriched in the LCMV Z VLP dataset (Fig 3A). For the intracellular interactome, however, several protein classes only met the high stringency cutoff for LASV Z (Fig 3B).

The various gene functional classification tools that exist can be useful for categorizing groups of proteins in proteomics or genomics datasets but a key limitation of these tools is the curation of gene ontology terms. For example, of the nine ESCRT proteins identified in LASV VLPs, only five of those proteins were included in the ESCRT-related functional class generated by the DAVID gene functional annotation tool (S1 and S2B Tables). Since the objective of this study was to better understand the molecular linkage between the PPXY late domain in Z and the cellular ESCRT pathway, classes of proteins which might provide a logical link to ESCRT proteins were curated. One possibility considered was that the cellular partners of Z themselves may contain late domains which could provide a direct or indirect linkage to ESCRT proteins. The ScanProsite tool was used to search for P(S/T)AP, YPX_(1,3)_L and PPXY domains across the human cellular proteome (S3A, S3B and S3C Table) [80, 81]. Another possibility considered was that the PPXY late domain in the Z protein recruits proteins containing WW domains. Lists of human cellular proteins containing WW domains based on Sudol and colleagues [82] as well as a comprehensive list of ESCRT proteins based on the recent review by Olmos and Carlton [14] were curated (S3D and S3E Table). These different protein classes were cross-referenced with the Z proteomics data (S1 Table), then odds ratio confidence intervals were calculated to determine the compatible range of enrichment of ESCRT proteins (Fig 3C) or proteins containing PPXY (Fig 3D), P(S/T)AP (Fig 3E), YPX_(1,3)_L (Fig 3F), or WW domains (Fig 3G), in any of the Z interactomes. ESCRT proteins were strongly enriched within the LCMV and LASV Z VLP and intracellular interactomes as well as within LCMV virions (Fig 3C), with odds ratio estimates ranging from 7 to 22. Proteins containing PPXY or P(S/T)AP domains were not significantly enriched in any of the conditions (Fig 3D and 3E). In the LASV Z intracellular interactome, the enrichment of YPX_(1,3)_L domains was statistically significant (p-value 0.004), but the odds ratio was modest (1.53) (Fig 3F). WW domain proteins, however, were enriched in the Z interactomes with odd ratios ranging from 3.92 to 6.82 (Fig 3G). The odds ratio for enrichment of WW domains in the intracellular LASV Z interactome was a moderate 3.92, and while the p-value for enrichment was 0.053 (losing significance after adjusting for multiple comparisons), since only 54 WW proteins are annotated in the human genome, all of the WW domain confidence intervals were large. Of these WW domain proteins, the Nedd4 family E3 ubiquitin ligases ITCH, WWP1 and WWP2 consistently co-purified with intracellular LCMV and LASV Z (Fig 2 and S1 Table) while Nedd4 only co-purified with intracellular LCMV Z in one replicate experiment (S1 Table). This is consistent with the findings of Fehling and Strecker [83] who showed that Nedd4 interacts with LASV Z via its PPXY late domain and demonstrates that additional proteins in this family interact with the Z protein.

### Nedd4 family E3 ubiquitin ligases interact with LCMV Z via its PPXY late domain

**Fig 4:**
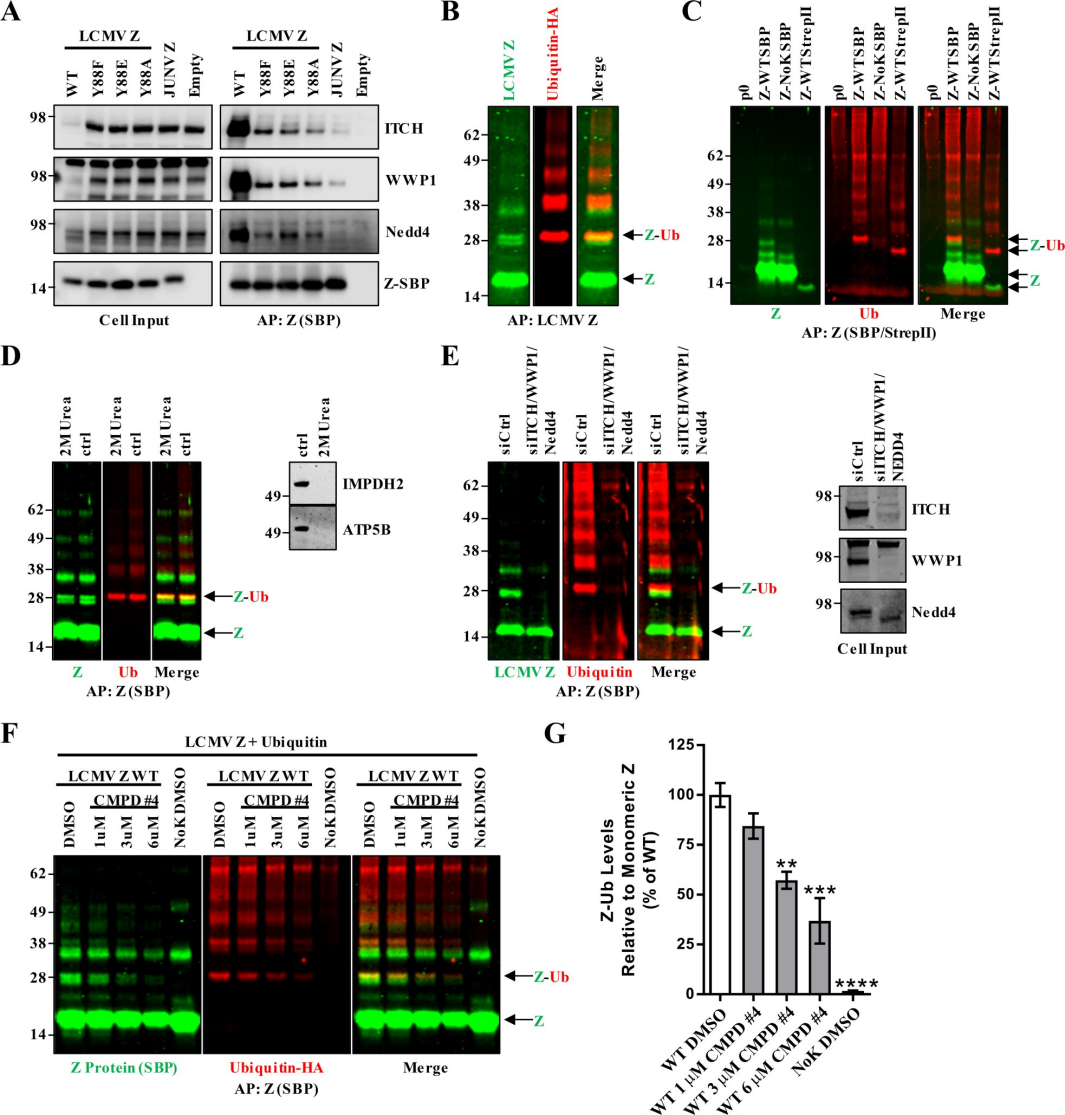
The LCMV Z protein is directly ubiquitinated on lysine residues by Nedd4 family ubiquitin ligases. (A) SBP-tagged LCMV Z (WT or Z with the tyrosine (88) in the PPXY late domain mutated), Junín virus (JUNV) Z, or an empty vector were expressed via plasmid in HEK293T cells. Z was affinity purified with streptavidin-coated magnetic beads and Z along with the Nedd4 family ubiquitin ligases ITCH, WWP1, and Nedd4 were detected via western blot. (B) SBP-tagged LCMV Z and hemagglutinin (HA)-tagged ubiquitin were expressed via plasmid in HEK293T cells and two days later Z was affinity purified (AP) with streptavidin beads and both Z and ubiquitin (Ub) were detected via two color fluorescent western blotting using anti-SBP and anti-HA antibodies. The Z monomeric band is indicated as well as overlapping bands at ~32kDa which may represent an ubiquitinated Z species (Z-Ub). (C) To distinguish between ubiquitinated host proteins and ubiquitinated Z, plasmids expressing either WT or lysine-free (NoK) LCMV Z with affinity tags of different molecular weights (SBP or Strep tag II) were co-transfected with HA-tagged ubiquitin in HEK293T cells and two days later Z was affinity purified with streptavidin beads and both Z and ubiquitin were detected via two color fluorescent western blotting using a combination of anti-SBP, anti-Strep tag II, and anti-HA antibodies. (D) In order to eliminate non-covalently interacting host proteins from co-purifying with LCMV Z, urea was added to the cell lysate to a concentration of 8M and incubated for 1 hour. The samples were then diluted with lysis buffer to reduce the urea concentration to 2 M and a streptavidin purification was performed. Western blotting was used to detect Z and ubiquitin as well as the known LCMV Z host partners IMPDH2 and ATP5B. (E) HEK293T cells were seeded then one day later small interfering (si)RNAs targeting ITCH, Nedd4, and WWP1 or a concentration of a non-targeting siRNA equal to the total of the three distinct siRNAs were transfected into the cells and two days later the cells were transfected with plasmids expressing SBP-tagged LCMV Z and HA-tagged ubiquitin. One day after the plasmid transfection, the cells were lysed and Z was affinity purified with streptavidin beads and was detected along with ubiquitin by two color fluorescent western blotting. (F-G) HEK293T cells were transfected with plasmids expressing LCMV Z WT or lysine-free Z (NoK) and HA-ubiquitin and treated with the indicated concentrations of the PPXY-Nedd4 interaction inhibitor, compound #4. Two days later the cells were collected and Z was purified with streptavidin beads from cell lysates and ubiquitin and Z were detected by two color fluorescent western blotting. (G) The quantity of the Z-Ub bands in the fluorescent western blots in (F) were quantified using Licor Image Studio software and divided by the quantity of monomeric Z from three independent experiments for all conditions except the 6μM and NoK DMSO conditions which were included only twice. Data in (F) represent the mean ± SEM of these experiments and a one-way ANOVA with Holm-Sidak’s test for multiple comparisons was used to compare the mean values to the WT DMSO condition. **p< 0.01, ***p< 0.001, ****p< 0.0001. Data in (A and F) are representative of two independent experiments and (B, D and E) are representative of at least three independent experiments.

To confirm Z’s interaction with these Nedd4 family ubiquitin ligases and determine if binding is dependent on Z’s PPXY late domain, we expressed three Z constructs via plasmid in HEK293T cells; WT LCMV Z, LCMV Z in which the tyrosine of the PPXY motif had been mutated (Y88F, Y88E, Y88A), and as a negative control, Z from the New World mammarenavirus JUNV. In our recent study of the JUNV Z interactome, no Nedd4 family ubiquitin ligases were identified likely due to the fact that JUNV Z encodes only a P(S/T)AP late domain and not the PPXY late domain found in Old World mammarenaviruses [57]. Affinity purification followed by SDS-PAGE and western blotting confirmed that ITCH, WWP1 and Nedd4 all co-purified with WT LCMV Z, while the interaction of LCMV Z proteins lacking intact PPXY late domains with these Nedd4 family proteins was drastically reduced (Fig 4A). As expected, JUNV Z also failed to efficiently co-purify with any of the Nedd4 proteins (Fig 4A). Interestingly, the levels of ITCH, WWP1, and Nedd4 in the cell input were reduced in the WT condition relative to Y88-mutant Z (Fig 4A). This could at least partially be explained by Z-mediated recruitment of these Nedd4 proteins into VLPs (S1 Table). Collectively, our results confirm that LCMV Z’s interaction with Nedd4 family proteins requires an intact PPXY late domain as has been shown for a range of other viruses including LASV [2, 83].

### LCMV Z is ubiquitinated by Nedd4 family E3 ubiquitin ligases

Nedd4 family proteins are E3 ubiquitin ligases that catalyze the final step in ubiquitin conjugation to substrate proteins [84]. Accordingly, the interaction of LCMV Z with Nedd4 family proteins could result in ubiquitination of Z. In order to test this, plasmids expressing SBP-tagged LCMV Z and hemagglutinin (HA)-tagged ubiquitin were co-transfected into HEK293T cells. Z was affinity purified with streptavidin beads and then Z and ubiquitin were detected by two-color fluorescent western blotting. For both Z and ubiquitin, there were several prominent bands of different molecular weights (Fig 4B). While SBP-tagged LCMV Z has an approximate molecular weight of 16 kDa, Z has been shown to form oligomers that may be represented by some of the higher molecular weight bands on the Z western blot (Fig 4B) [85, 86]. Notably, the dimeric form of Z has been shown to exhibit resistance to denaturing conditions previously [85]. However, a band of protein near 32 kDa was present in both the Z and ubiquitin channels that could correspond to a Z protein that is modified with two ~8 kDa ubiquitin molecules (Fig 4B). The conditions under which the affinity purification was performed for Fig 4B were equivalent to those used in the initial proteomics screen, leaving open the possibility that the ubiquitinated protein represented by the band at 32 kDa in Fig 4B could represent a ubiquitinated host protein that interacts non-covalently with LCMV Z and migrates to an identical position during gel electrophoresis. To rule out this possibility, a plasmid expressing WT LCMV Z with a smaller, Strep tag II affinity tag was transfected into HEK293T cells with HA-tagged ubiquitin as above and compared to SBP-tagged WT or a lysine-free (NoK) LCMV Z (Fig 4C). A molecular weight shift is readily observed in the red channel (HA-ubiquitin) with the StrepII-tagged Z relative to the SBP-tagged Z that correlates with the molecular weight differences conferred by the affinity tags (Fig 4C). Additionally, some higher molecular weight bands in the red channel also appear to be shifted in size and thus may also correspond to ubiquitinated Z (Fig 4C). Notably, mutation of all lysine residues in the NoK LCMV Z mutant eliminated the majority of the ubiquitin signal which not only suggests Z is directly ubiquitinated but that the modification occurs at one or more lysine residues, which is the amino acid that ubiquitin is most often conjugated to (Fig 4C) [87]. Finally, as a complementary approach, the affinity purification was repeated with 2 M urea (Fig 4D). The urea treatment eliminated the interaction between Z and the cellular proteins IMPDH2 and ATP5B, which were previously shown to interact with LCMV Z [57], but the Z-ubiquitin (Ub) species was retained. Collectively, these data strongly suggest that Z itself is ubiquitinated on lysine residues.

As Nedd4 family E3 ubiquitin ligases interact with LCMV Z in a PPXY-dependent manner, we hypothesized that these ligases could be responsible for the observed ubiquitination of Z. To further test this hypothesis, we used small interfering (si)RNAs to simultaneously knockdown ITCH, Nedd4 and WWP1, and saw that the loss of these three proteins resulted in the loss of ubiquitinated Z (Fig 4E). As it appeared that ubiquitination of Z was dependent on both the viral late domain and the cellular Nedd4 family E3 ligases, we attempted to perturb this interaction by a complementary method. A small molecule (compound #4) has recently been by described by Harty and colleagues that inhibits the PPXY-Nedd4 interaction [88]. Accordingly, we treated Z-SBP and HA-ubiquitin-transfected cells with compound #4. We observed that increasing concentrations of compound #4 resulted in reduced levels of Z-Ub, in a dose-dependent manner (Fig 4F and 4G). Collectively, these results strongly suggest that Nedd4 family E3 ubiquitin ligases are responsible for the ubiquitination of LCMV Z.

### The ubiquitin-binding protein HGS/Hrs is required for the release of LCMV DI and infectious particles

**Fig 5:**
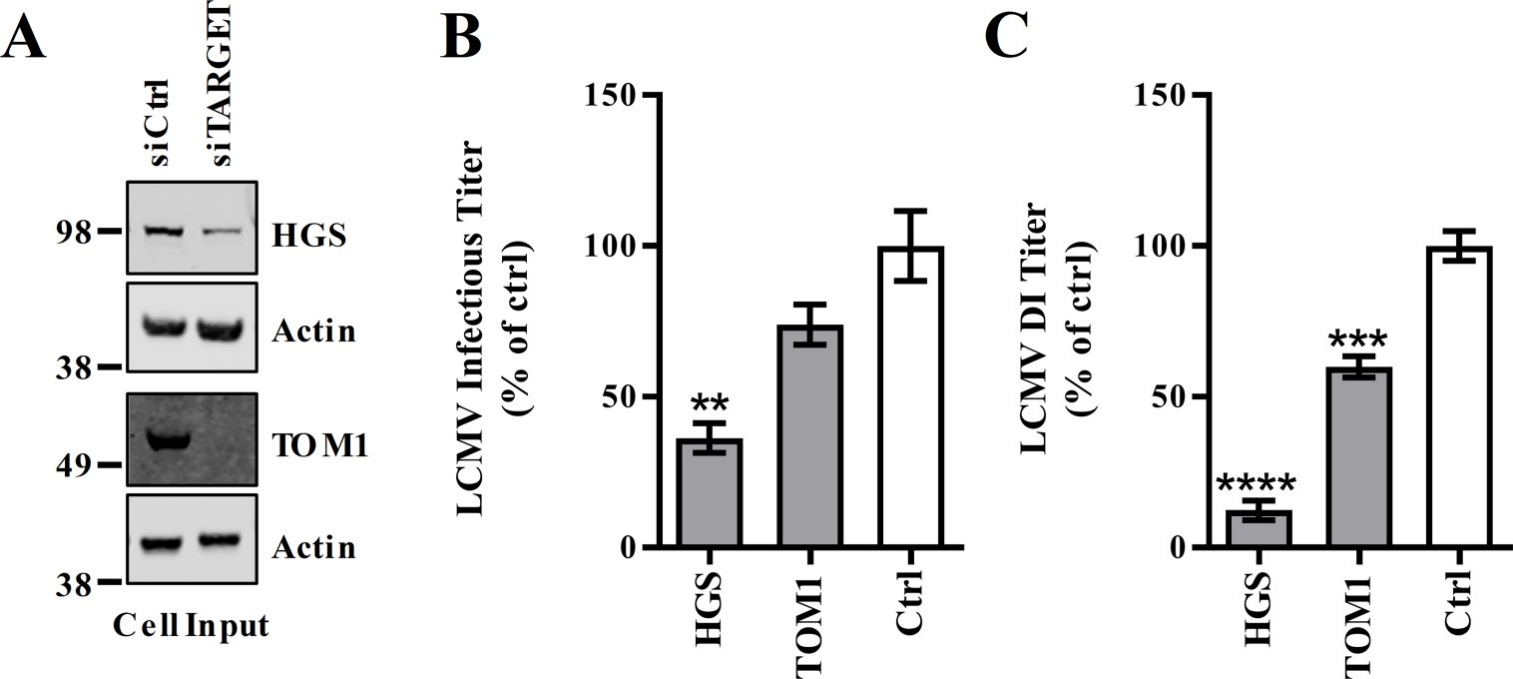
The ubiquitin-binding, ESCRT-0 protein HGS is required during mammarenavirus infection. (A-C) Human lung carcinoma (A549) cells were reverse transfected with small interfering (si)RNAs to the indicated targets or a non-targeting control siRNA and then two days later were infected with LCMV and cells and virus-containing cell culture media were collected after an additional two days. (A) Expression of siRNA-targeted proteins and actin was measured in cells by western blot. Infectious titers were determined by plaque assay (B) and LCMV DI particle titers were measured by plaque interfering assay (C). Data represent the mean ± SEM of three independent experiments. A one-way ANOVA with Holm-Sidak’s test for multiple comparisons was used to compare the mean values to the control siRNA in (B-D). **p< 0.01, ***p< 0.001, ****p< 0.0001.

If the role of Z’s PPXY late domain is to recruit Nedd4 family ubiquitin ligases in order to mediate protein ubiquitination, presumably there are ubiquitin-binding proteins that can recognize these ubiquitinated substrates and link viral proteins to the ESCRT pathway. Accordingly, the Z interactome was searched for ubiquitin binding proteins. Many of the proteins found were ESCRT proteins including the ESCRT-I proteins MVB12A, UBAP1 and Tsg101 as well as the ESCRT-accessory protein ALIX (official gene symbol PDCD6IP) (S1 Table). The ESCRT-0 protein Hrs (official gene symbol HGS), which has a well-defined role in recognizing ubiquitinated proteins, also consistently interacted with intracellular LCMV and LASV Z (S1 Table) [89]. In addition, the ESCRT-0-like protein, TOM1, was part of the LCMV Z intracellular interactome (Fig 2 and S1 Table). To determine whether any of these ubiquitin-binding proteins are involved in LCMV infection, the expression of HGS or TOM1 were knocked down using siRNAs (Fig 5A). HGS knockdown resulted in a significant reduction in LCMV infectious release (Fig 5B) while production of DI particles was inhibited to a greater extent (Fig 5C). Loss of TOM1 also resulted in a modest, but statistically significant decrease in LCMV DI particle production (Fig 5C).

### LCMV Z is ubiquitinated at K10 and K77

**Fig 6:**
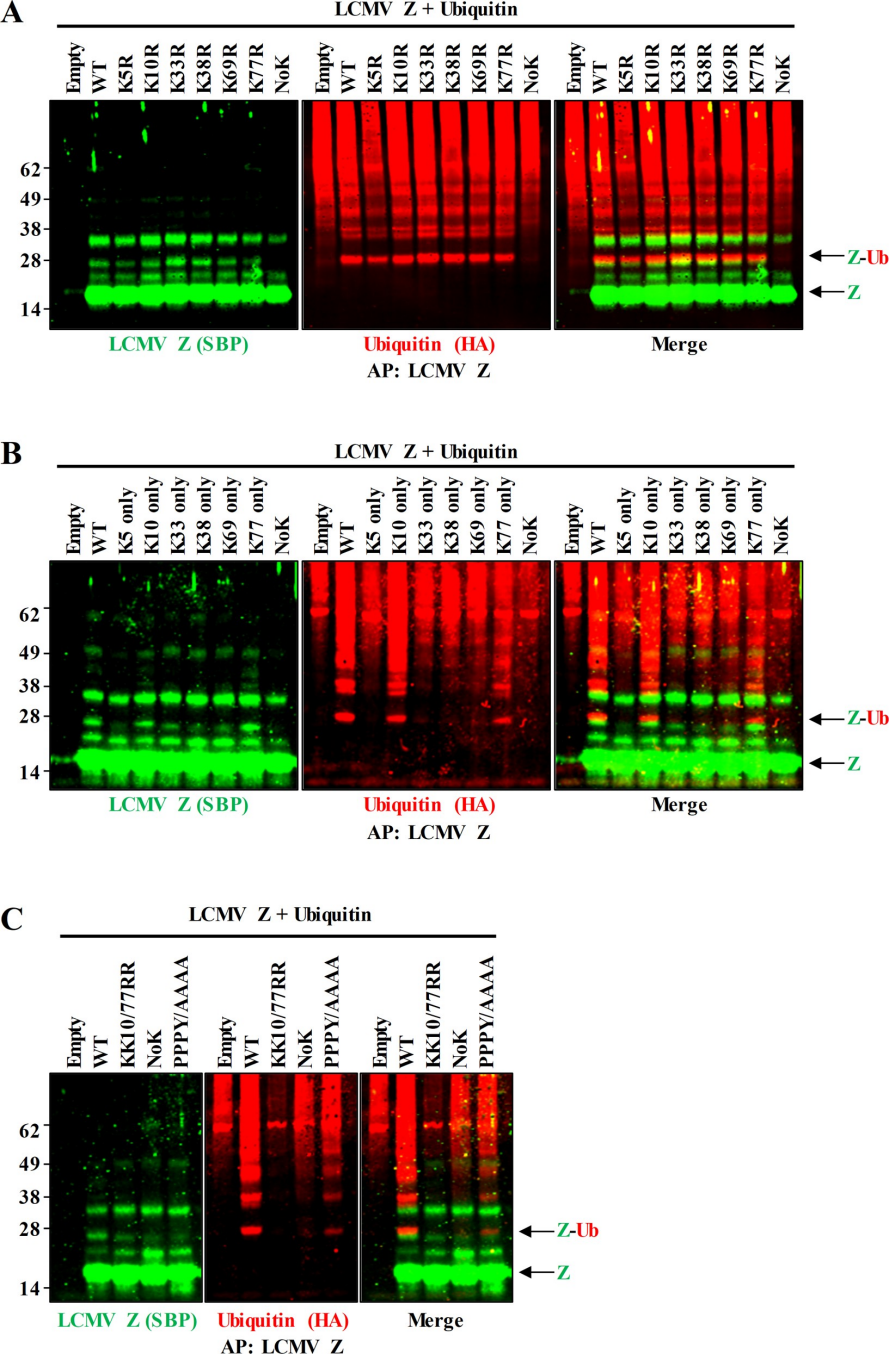
The LCMV Z protein is ubiquitinated at lysines 10 and 77. (A) Plasmids expressing SBP-tagged LCMV Z WT, Z containing lysine to arginine mutations at each of the six lysine residues, Z-NoK, or an empty vector were transfected into HEK293T cells along with a plasmid expressing HA-tagged ubiquitin (HA-Ub) and then two days later Z was affinity purified and was detected with ubiquitin by two color western blotting. (B) Plasmids expressing HA-Ub and LCMV Z or an empty vector were transfected into HEK293T cells, affinity purified, and detected via western blotting as in (A), except the lysine mutant Z constructs that were used each contained only the one intact lysine residue indicated, or no intact lysines (NoK). (C) Plasmids expressing HA-Ub and an empty vector or LCMV Z WT, LCMV Z mutant with both lysines 10 and 77 mutated to arginine, LCMV Z-NoK, or a LCMV Z PPXY late domain mutant (PPXY/AAAA) were transfected into HEK293T cells, affinity purified, and detected as in (A-B). Each panel is representative of at least 3 independent experiments.

To further confirm that LCMV Z is directly ubiquitinated, the prediction tool, UbPred, was used to predict possible sites of ubiquitination on LCMV Z [90]. The lysine residues at position 5 and 10 were the only residues predicted to be ubiquitin acceptor sites. However, LCMV Z constructs with these lysine residues mutated to arginine, to block ubiquitin conjugation [87], had similar levels of the 32 kDa Z and ubiquitin bands present relative to WT Z (Fig 6A). As mutation of the two lysine residues predicted to be ubiquitinated in LCMV Z did not alter Z-Ub levels, it was possible that one of the four non-predicted lysine residues were ubiquitinated. While mutating all lysines in LCMV Z blocked ubiquitination of Z, no individual lysine mutation resulted in decreased levels of Z-Ub (Fig 6A). This result suggested that more than one lysine residue in Z can be ubiquitinated. To determine which of the six lysine residues in Z can serve as ubiquitin acceptor sites, a panel of LCMV Z plasmids in which only one of the six lysine residues in each Z plasmid was intact was generated (Fig 6B). This revealed that lysine 10 (K10) and lysine 77 (K77) are the only two lysine residues in Z that can be efficiently ubiquitinated (Fig 6B). Additionally, the Z-Ub band observed in the western blot in the individual K10 and K77 mutants maintained a molecular weight of ~32 kDa, co-migrating with the WT band (Fig 6B). This suggests that in WT virus, either K10 or K77 is modified by di-ubiquitin rather than each of the residues being mono-ubiquitinated. Finally, to confirm that K10 and K77 are the only potential ubiquitination sites in LCMV Z, a double mutant was generated. This KK10/77RR mutant blocked ubiquitination of Z as effectively as the LCMV Z-NoK mutant (Fig 6C). Finally, LCMV Z lacking an intact late domain (PPPY to AAAA) had significantly reduced levels of Z-Ub, supporting a role for Nedd4 ligases in Z ubiquitination (Fig 6C). However, the levels were not reduced to the extent that the lysine mutants were, suggesting that these ligases may be able to ubiquitinate Z, albeit less efficiently, in the absence of a fully intact PPXY late domain (Fig 6C).

### Direct ubiquitination of LCMV Z is not required for virus particle release

**Fig 7:**
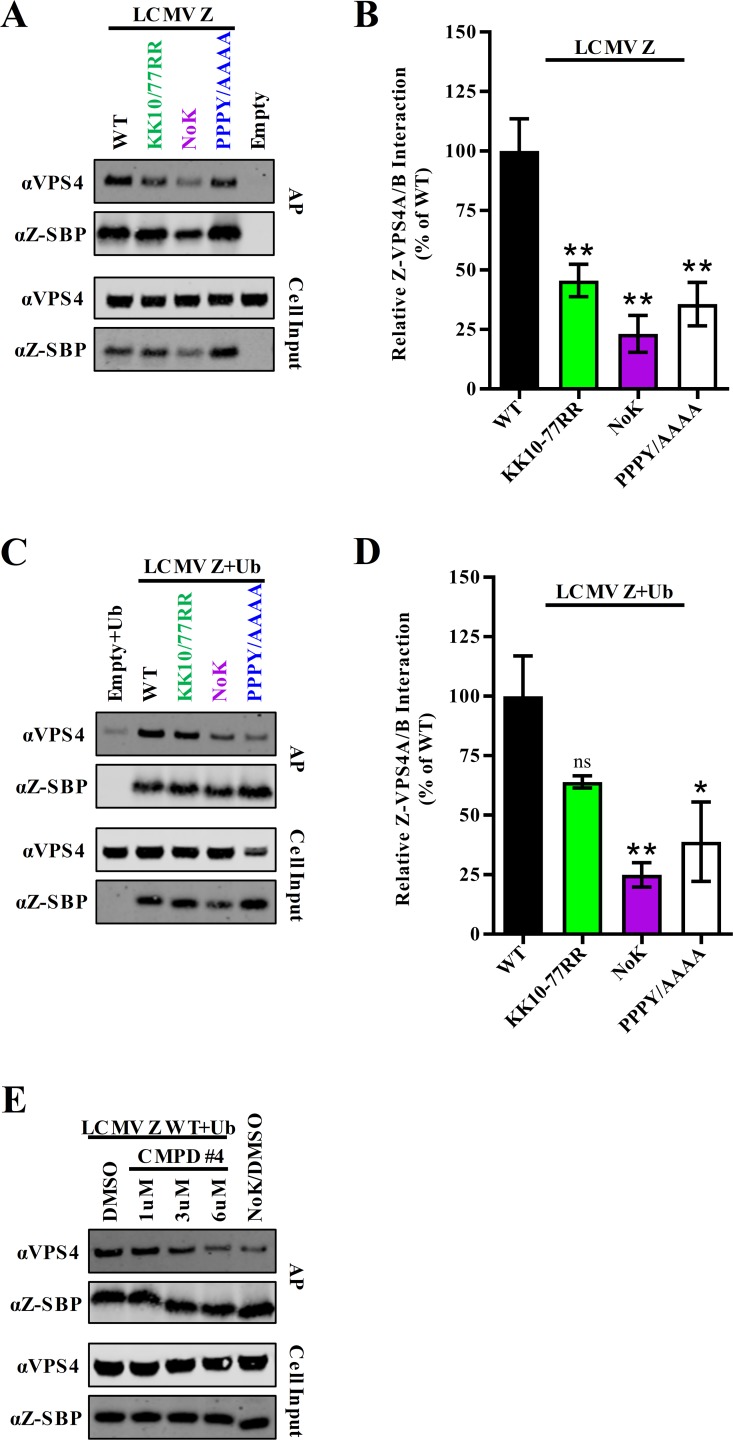
Efficient interaction of LCMV Z with the ESCRT protein VPS4 requires residues K10, K77, and the PPXY late domain. (A-B) HEK293T cells were transfected with an empty vector or a vector encoding SBP-tagged LCMV Z with the indicated mutations. Z was affinity purified using streptavidin beads and the purified Z (bait) and VPS4 (prey) were detected by western blotting. (B) The quantity of co-purified VPS4 in the fluorescent western blots in (A) was quantitated using Licor Image Studio software and divided by the quantity of affinity purified monomeric Z. (C-D) HEK293T cells were transfected with plasmids expressing HA-tagged ubiquitin and either an empty vector or a vector encoding SBP-tagged LCMV Z with the indicated mutations. Z was affinity purified using streptavidin beads and the purified Z (bait) and VPS4 (prey) were detected by western blotting as described in (A). (D) The quantity of co-purified VPS4 in the fluorescent western blots in (C) was quantified using Licor Image Studio software and divided by the quantity of affinity purified monomeric Z. (E) HEK293T cells were transfected with plasmids expressing HA-tagged ubiquitin and SBP-tagged WT or NoK LCMV Z and treated with the indicated doses of compound #4 or DMSO vehicle control. Z was affinity purified after two days and Z and VPS4 were detected by western blotting. The western blots shown are representative of three independent experiments. The graphs in (B) and (D) represent the mean values ± SEM of three independent experiments. A one-way ANOVA with Holm-Sidak’s test for multiple comparisons was used to compare the mean values to the WT control in (B) and (D). *p< 0.05, **p< 0.01.

Several approaches were used to assess the importance of Z ubiquitination for LCMV propagation. First, the ability of a panel of LCMV Z mutants to interact with the ESCRT protein VPS4 was assessed by affinity purification and western blotting (Fig 7A). By mass spectrometry, several ESCRT factors were identified as partners of LCMV Z including the ATPases VPS4A and VPS4B which are required for disassembling the ESCRT machinery at sites of membrane scission (Fig 2 and S1 Table) [91]. As detection of endogenous ESCRT proteins by western blot remains challenging, we chose to focus on VPS4A/B, as we can reliably detect endogenous levels of the protein. LCMV Z that cannot be ubiquitinated (KK10/77RR) was deficient in its ability to interact with VPS4A/B (Fig 7A and 7B). Following affinity purification, the levels of VPS4A/B normalized to levels of affinity purified Z were 54% less compared to WT Z (Fig 7A and 7B). This is nearly as great of a reduction as observed with the PPPY late domain mutant, which bound 64% less VPS4A/B compared to WT Z (Fig 7A and 7B). An even more substantial defect was observed with the lysine-free LCMV Z mutant (NoK) (Fig 7A and 7B). While a conservative substitution to arginine was used, we cannot rule out that these mutants have structural defects. Overexpression of ubiquitin was able to modestly compensate for the loss of the identified ubiquitin acceptor sites (KK10/77RR), but did not appear to impact the interaction of lysine-free or PPXY-mutant Z with VPS4A/B (Fig 7C and 7D). We also assayed Z-VPS4 binding in transfected cells following treatment with compound #4 to disrupt PPXY-Nedd4 interactions and as expected we saw a dose-dependent decrease in the Z-VPS4 interaction (Fig 7E). Together these results suggest that ubiquitination of Z may play a role in ESCRT recruitment but, importantly, that non-ubiquitinated Z can interact with VPS4A/B in the absence of a ubiquitin modification and may suggest that ubiquitination of trans-acting cellular proteins may also be involved.

**Fig 8:**
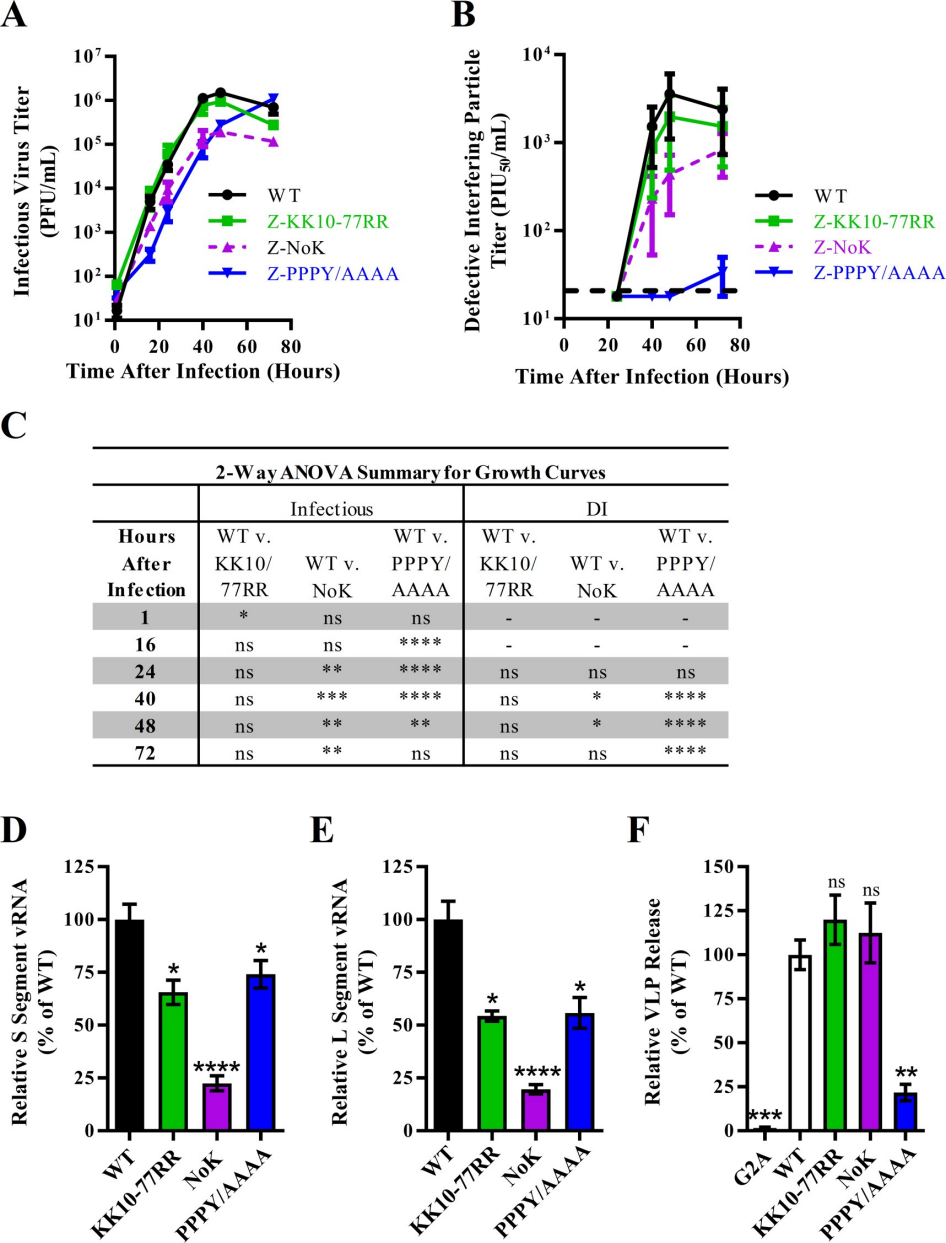
Loss of ubiquitination sites in LCMV Z has little impact on virus particle release. (A-B) Reverse genetics was used to generate recombinant (r)LCMV containing mutations at the two ubiquitination sites (K10 and K77) as well as a virus with a lysine-free (NoK) or a late domain-free (PPPY>AAAA) Z protein. The growth kinetics of these viruses were then measured by infecting A549 cells with these viruses and measuring the infectious titers by plaque assay (A) and DI particle titers by plaque interfering assay (B) at 1, 16, 24, 40, 48 and 72 hours after infection. (C) Summary of a two-way ANOVA with Holm-Sidak’s test for multiple comparisons used to compare the log-transformed mean titer values from the growth curve in (A-B). (D-E) Viral RNA was isolated from clarified cell culture media from the 72 hours post-infection time point in (A-B) and the quantities of genomic S-segment (D) or L-segment (E) vRNA were determined by quantitative real time RT-PCR. (F) The release of virus-like particles (VLPs) from HEK293T cells transfected with plasmids expressing SBP-tagged LCMV Z protein with the indicated mutations was measured. The graphs in (A-B and D-F) represent the mean values ± SEM of three independent experiments with two technical replicates each. A one-way ANOVA with Holm-Sidak’s test for multiple comparisons was used to compare the mean values to the WT control in (D-F). *p< 0.05, **p< 0.01, ***p< 0.001, ****p< 0.0001.

To test the role of Z ubiquitination during infection, recombinant (r)LCMVs containing the designated mutations were generated and their growth kinetics were assessed by a multistep growth curve in A549 cells (Fig 8A and 8B). Release of both infectious and defective interfering (DI) virus particles for KK10/77RR rLCMV was not significantly different from WT virus (Fig 8A, 8B and 8C). To ensure that the near WT phenotype of the KK10/77RR mutant was not due to a reversion or acquisition of a compensatory mutation, both genome segments were sequenced but no changes were identified in any of the four open reading frames. Production of both infectious and DI particles was significantly reduced for rLCMV containing lysine-free Z-NoK (Fig 8A, 8B and 8C). Growth kinetics of the late domain mutant rLCMV (PPPY/AAAA) were similar to that of the PPPY/PPPF mutant previously published which produced little or no DI particles (Fig 8A, 8B and 8C) [76]. Quantities of each viral genome segment were measured in the 72 hours post infection growth curve samples by quantitative PCR (Fig 8D and 8E). While there was not a statistically significant decrease in either infectious or DI particles (Fig 8A and 8B), a statistically significant but modest decrease in S- and L-segment genome copy numbers in the supernatant was observed for viruses containing either the double mutant (KK10-77RR) or the mutated late domain, and this defect was even larger for the lysine free virus (Fig 8D and 8E). Finally, a virus-like particle (VLP) release assay described previously [76, 92] was used to measure the contribution of Z ubiquitination to virus release (Fig 8F). As expected, the G2A mutant, which cannot be myristoylated, had significantly reduced levels of VLP release (Fig 8F). No difference between WT and Z-KK10/77RR or the lysine free mutant were observed, while the PPPY late domain mutation significantly reduced VLP release (Fig 8E). Together these results suggest that direct ubiquitination of Z has little impact on the ability of LCMV Z to drive budding in a VLP assay or for intact LCMV to successfully complete its life cycle.

### ITCH and WWP1 are critical for LCMV release

**Fig 9:**
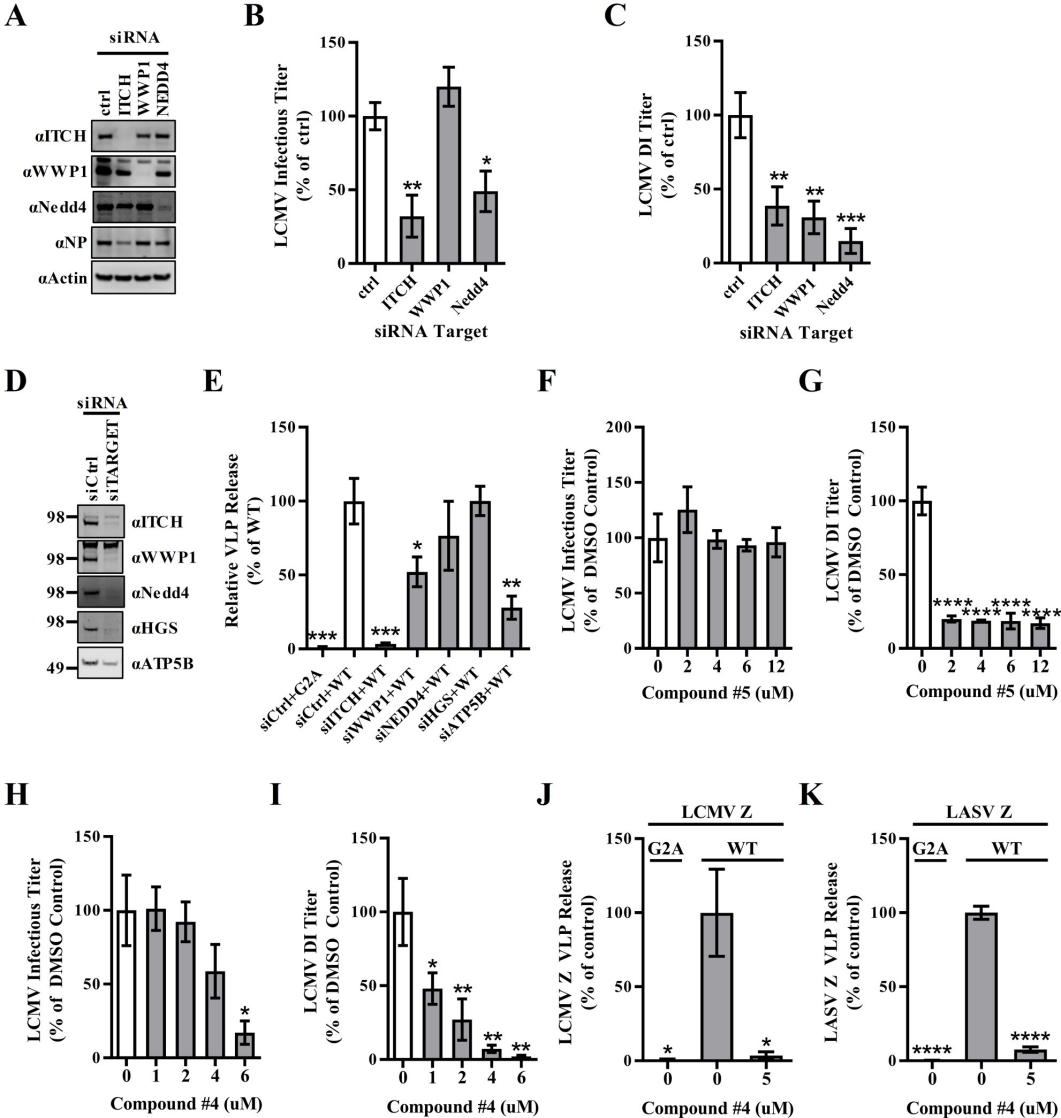
ITCH and WWP1 are required for virus release. (A-C) Human lung carcinoma (A549) cells were reverse transfected with small interfering (si)RNAs to the indicated targets or a non-targeting control siRNA then two days later were infected with LCMV and cells and virus-containing cell culture media were collected after an additional two days. (A) Expression of siRNA-targeted proteins, the viral nucleoprotein (NP), and actin was measured in cells by western blot. Infectious titers were determined by plaque assay (B) and LCMV DI particle titers were measured by plaque interfering assay (C). (D-E) HEK293T cells were reverse transfected with siRNAs to the indicated targets or non-targeting control siRNA then two days later were transfected with a plasmid expressing SBP-tagged WT LCMV Z. (D) Expression of siRNA-targeted proteins was measured in cells by western blot and (E) the quantity of Z in cells and VLPs was determined by quantitative western blotting from which the percent VLP release was determined. (F-I) HEK293T cells were infected with LCMV and then treated after 1 hour with compound #5 (F-G) or #4 (H-I). Infectious titers and DI particle titers were measured from the cell culture supernatant collected two days after infection. (J-K) Release of VLPs from LCMV (J) or LASV (K) Z-transfected HEK293T cells treated with compound #4 was measured by quantitative western blotting. A one-way ANOVA with Holm-Sidak’s test for multiple comparisons was used to compare the mean values to the control siRNA (B, C and E) or the DMSO control (F-K). Data in (B-C and E-K) represent the mean ± SEM from four (B-C) or three (E-K) independent experiments. *p< 0.05, **p< 0.01, ***p< 0.001, ****p< 0.0001.

While the data presented here suggest that ubiquitination of LCMV Z by Nedd4 family proteins appears to be dispensable for LCMV release, Z’s PPXY late domain specifically recruits these ligases (Fig 4A and [83]) and is required for DI particle release [76]. In order to determine whether these ligases are functionally required for efficient virus particle release, ITCH, WWP1, or Nedd4 expression was knocked down with siRNA and cells were challenged with LCMV (Fig 9A). Two days after virus inoculation, depletion of ITCH or Nedd4 significantly reduced release of both infectious and DI LCMV particles (Fig 9B and 9C). In contrast, knockdown of WWP1 specifically reduced the production of LCMV DI particles, but not did not affect production of infectious virions (Fig 9B and 9C). Because these infections were initiated using a low MOI of 0.001 PFU/cell, the assay cannot specifically define the virus life cycle stage that is impacted. To determine whether any of the Nedd4 ubiquitin ligases impact virus release specifically, a VLP release assay was employed which revealed that ITCH and WWP1, but not Nedd4 or HGS, are required for virus release (Fig 9D and 9E).

As a complementary approach, LCMV infected cells were treated with the PPXY-Nedd4 inhibitor compound #4, and a related compound #5, both developed by Harty and colleagues [88]. Compound #5 reduced LCMV DI particle titers but did not impact infectious virus (Fig 9F and 9G) similar to the phenotype observed with WWP1 knockdown (Fig 9B and 9C). Alternatively, both infectious and DI particle titers were reduced by compound #4 in a dose dependent manner, though DI particle titers were more severely impacted (Fig 9H and 9I). A VLP release assay was also employed demonstrating the compound #4 inhibits both LCMV and LASV Z VLP release (Fig 9J and 9K), which agrees with work by Harty and colleagues [88]. Combined, these data support a role for Nedd4 family ligases in facilitating the release of LCMV virus particles, likely through ubiquitination of trans-acting host proteins or possibly other viral proteins. Furthermore, these data raise the possibility that certain Nedd4 family proteins are involved in the PPXY-mediated ESCRT recruitment required specifically for DI particle release, as observed for WWP1. Other Nedd4 family members appear to act more broadly and, despite interacting with LCMV Z in a PPXY-dependent manner, do not impact infection in the same way.

## Discussion

In this study, we sought to use LCMV as a model to better understand how viruses use the PPXY late domain to recruit the ESCRT pathway to mediate virus release. The proteomics study conducted here identified four Nedd4 family ubiquitin ligases as partners of the Z protein. We tested the hypothesis that viral PPXY late domains recruit ESCRT proteins by Nedd4-mediated ubiquitination of the viral protein containing the late domain. We demonstrate that these ligases ubiquitinate LCMV Z and pinpoint the two lysine residues in LCMV Z that are targeted. Mapping the exact ubiquitination sites permitted us to interrogate the impact of direct ubiquitination of Z during infection, without perturbing cellular pathways, something that has been done for few other viruses. Our data suggest that direct ubiquitination of Z is dispensable for virus release and other functions but, importantly, supports the hypothesis that Nedd4 family ubiquitin ligases are critical during infection. WWP1 specifically appears to impact the release of LCMV DI particles suggesting that it is a critical part of the link between the PPXY late domain in Z and the ESCRT pathway, both of which drive DI particle release. Furthermore, several partners of LCMV and LASV Z identified here have plausible biology for involvement with the ESCRT pathway.

Nedd4 family ubiquitin ligases have been implicated in the release of viruses with PPXY late domains since the matrix proteins of rabies, vesicular stomatitis, and Ebola viruses were shown to interact with Nedd4 [39, 93]. For many viruses with PPXY late domains, these ligases are known to be required for efficient virus release and several studies have underscored the importance of their ligase activity in this process [2]. It is perhaps unsurprising, then, that we identified several proteins in this family in both the LCMV and LASV Z interactome. Indeed, a PPXY-dependent LASV Z-Nedd4 interaction has been shown previously [83], though, Nedd4 specifically does not appear to be required for LASV VLP release [94]. The data presented here, combined with work by Harty and colleagues with LASV VLP release, suggest at least some of these ligases are required during Old World mammarenavirus release [88]. However, how these ligases contribute to mammarenavirus release, including whether ubiquitination is involved and, if so, what the targets of ubiquitination pertinent to virus release are, remain unknown. Beyond mammarenaviruses, there is evidence that Ebola and Marburg virus VP40 and some retroviral Gag proteins are ubiquitinated by Nedd4 family ligases, but with the exceptions of human T-cell leukemia virus type 1 (HTLV-1) and murine leukemia virus (MLV) Gag [43, 95], the specific residues that are modified have not been identified. This has prevented understanding how direct ubiquitination of these viral budding proteins contributes to virus release.

We reasoned that ubiquitin could be used by PPXY-containing viral budding proteins in a manner equivalent to that used in trafficking of integral plasma membrane receptor proteins through the endosomal pathway. In this model, signaling from integral plasma membrane receptors can be down-regulated by endocytosis of the receptor itself [96]. While these endosomes and their receptor cargo can recycle back to the plasma membrane, the endocytosed receptor can also be ubiquitinated within its cytosolic domain [96]. This modification results in binding of ESCRT-0 proteins, the subsequent ESCRT-dependent formation of intraluminal vesicles containing the receptor, and the ultimate degradation of the receptor in the lysosome [96]. In this study, we have shown that only two out of the six lysine residues in the LCMV Z protein are ubiquitinated. K77 is the closest lysine residue to the late domain which fits with studies of other viruses showing that lysines close to the PPXY domain are ubiquitinated [95, 97, 98]. The N- and C-terminal domains of Z appear to be unstructured and quite flexible, which may allow K10, which lies within the C-terminal domain, to be closer to the PPXY domain, in three-dimensional space, than its position in the linear amino acid sequence may suggest [86, 99, 100]. While these ubiquitination sites in Z impacted the interaction of Z with the ESCRT protein VPS4A/B in the context of transfection (Fig 7), during infection with a complete virus, the impact of mutating these sites was minimal (Fig 8). These two lysine residues are quite poorly conserved among Old World mammarenaviruses [101]. In fact, only one lysine in Z is highly conserved among the Old World mammarenaviruses, K69 in LCMV Z, but it does not appear to be ubiquitinated in LCMV (Fig 6B). However, others have shown that the homologous lysine residue in LASV Z is required for efficient viral RNA synthesis [101]. This could explain why both infectious and DI particle production were significantly attenuated for rLCMV Z-NoK (Fig 8A and 8B) but release of VLPs for the NoK mutant was not (Fig 8F).

The results presented here with LCMV Z suggest that direct ubiquitination of PPXY-containing viral proteins may not alone be sufficient for ESCRT recruitment, a finding supported by other studies. Prototypic foamy virus harboring a chimeric Gag protein containing a PPXY domain is ubiquitinated at its only lysine residue, but this modification is dispensable for virus budding [98]. In MLV, a single lysine residue in the p12 portion of Gag is monoubiquitinated, but loss of this ubiquitination acceptor site did not affect viral replication, at least *in vitro* [95, 97]. In HTLV-1, mutation of the lone ubiquitin acceptor site in the MA protein significantly reduced release of infectious particles [43]. However, the defect was not as severe as that of the PPXY-mutant and budding of this ubiquitin site mutant was still susceptible to catalytically inactive WWP1 expression [43]. Similarly, overexpression of Nedd4 family ubiquitin ligases can enhance budding of PPXY-containing viruses [38, 50–52, 54, 102], but can also enhance budding of viruses lacking a PPXY domain [103–105].

Together, these data suggest that other proteins may be the relevant targets of ubiquitination and may serve as links to ESCRT proteins. The arrestin-related trafficking protein, ARRDC1, has been implicated in PPXY-dependent ESCRT recruitment by MLV [106]. The exact mechanism by which ARRDC1 contributes to ESCRT recruitment for MLV is unclear, but its PSAP domain and ubiquitination of ARRDC1 itself by WWP1, an interaction mediated by two PPXY domains in ARRDC1, are likely involved [106]. It is yet unclear whether ARRDC1 contributes to ESCRT recruitment by other PPXY-containing viruses. None of the arrestin related trafficking proteins were identified in the Z interactome or in LCMV virions, but this certainly does not rule out a role for these proteins in mammarenavirus release as the absence of these proteins in the Z interactome could be due to a failure of detection. Other cellular proteins containing a PPXY domain, however, were identified in the Z interactome. The ESCRT-0 protein HGS was a partner of both LCMV and LASV Z (Fig 2 and S1 Table). This fits with recent work showing that HGS is recruited into VLPs by the RSV PPXY late domain [107]. We have shown here that HGS is required for efficient LCMV propagation (Fig 5), but that it does not appear to impact virus release (Fig 9E). Finally, annexin A6 (ANXA6) and thrombospondin 1 (THBS1) were found in LCMV and LASV VLPs and in LCMV virions. These proteins have both a PPXY domain and a YPX_(1,3)_L domain, which could provide a link to the ESCRT pathway much like ARRDC1 has been proposed to do through ubiquitination or direct binding to an ESCRT protein [106].

In addition to cellular proteins with PPXY domains, other proteins may function in concert with Nedd4 family ubiquitin ligases or serve an auxiliary role in ESCRT recruitment. Numerous proteins were found containing P(S/T)AP or YPX_(1,3)_L late domains that, presumably, can interact directly with the ESCRT proteins Tsg101 or ALIX, respectively (S1 Table). While proteins with these domains were not, as a group, overrepresented in the interactome (Fig 3E and 3F), that does not negate the possibility that some of these proteins could provide a linkage between Z and ESCRT. Syntenin (SDCBP), for example, which functions as an ESCRT adaptor protein in exosome release [18], was identified in both the LCMV and LASV Z interactome in the second replicate experiment. CEP55, which serves as an ESCRT adaptor protein for cellular abscission through its unique, direct interaction with Tsg101, was also identified in the Z interactome [12]. Determining how these and other proteins identified here contribute to Z protein function may enhance our understanding of PPXY-mediated virus release.

Nedd4 family ubiquitin ligases play an important role in the release of a range of enveloped viruses, including both those with PPXY late domains and those without. In the case of viruses with PPXY late domains, our work, combined with that of others, makes it increasingly clear that trans-acting cellular proteins are critical in ESCRT-dependent virus release. ARRDC1 represents one such trans-acting protein, but perhaps as outlined here, additional cellular proteins may play a similar role. There are well over 1,000 cellular proteins that contain PPXY domains. Being likely targets of Nedd4 family-mediated ubiquitination, identifying the proteins within this group that are involved in virus release is critical. It is also worth considering how Nedd4 family proteins may contribute to stages of the life cycle other than release for different viruses. The regulation of interferon-induced transmembrane protein 3 (IFITM3) by Nedd4 represents such an example whereby Nedd4 induces degradation of IFITM3 through ubiquitination and promotes influenza virus infection [108]. Finally, it will be important to consider the relative contribution that ubiquitination of these trans-acting proteins plays in these processes. While ubiquitinated trans-acting proteins could bind ubiquitin binding domains in ESCRT proteins, ubiquitination-independent mechanisms could also be at work. Potentially, the chain of WW domains in Nedd4 family proteins could themselves link the viral PPXY domain with PPXY in a trans-acting protein [98, 106]. If this trans-acting protein is capable of interacting with ESCRT proteins, through P(S/T)AP, YPX_(1,3)_L, or other domains like that found in CEP55 or the Nipah virus C protein [12, 109], an ESCRT linkage could be made independent of ubiquitin. In order to tease out such mechanisms, it will be important to identify sites of Nedd4 family-mediated ubiquitination, as we have done here for LCMV Z, in order to separate the impact of ubiquitination of a single protein from the much broader effect these ligases have.

In conclusion, the current study advances our understanding of mammarenaviruses by showing that Nedd4 family ubiquitin ligases are required for the efficient release of LCMV DI particles. Surprisingly, direct ubiquitination of LCMV Z by Nedd4 family ubiquitin ligases was not required for LCMV particle release, suggesting that other viral and/or host proteins may serve as important targets of ubiquitination to link Z to the ESCRT pathway. This finding, which is in agreement with similar studies of MLV Gag [95] but not HTLV-1 [43], indicates that there is no clear, single mechanism at work to explain how Nedd4 family ubiquitin ligases contribute to the budding process of PPXY-containing matrix proteins. Indeed, viruses from different families, or even within a particular family, may differentially utilize Nedd4 family ligases to facilitate budding and possibly additional stages of the viral life cycle and it will be important to define the mechanism at work in each case. Notably, the human proteins mapped here as partners of Old World mammarenavirus matrix proteins or components of VLPs or virions will provide a framework for further investigation of the mechanisms driving mammarenavirus release as well as enabling a better understanding of other Z protein functions.

## Materials and methods

### Cells and viruses

Human embryonic kidney (HEK-293T/17) (CRL-11268), referred to as HEK293T here, and human lung carcinoma (A549) (CCL-185) cells were purchased from American Type Culture Collection (Manassas, VA). HEK293T cells were maintained in Dulbecco’s Modified Eagle Medium (DMEM) (11965–092) supplemented with 10% fetal bovine serum (FBS) (16140–071), 1% penicillin-streptomycin (15140–122), 1% MEM Non-Essential Amino Acids Solution (11140–050), 1% HEPES Buffer Solution (15630–130) and 1% Gluta-MAX (35050–061) purchased from Thermo Fisher Scientific (Carlsbad, CA). A549 cells were maintained in 50:50 DMEM:F-12 media (11330–032, Thermo Fisher Scientific) supplemented with 10% FBS and 1% penicillin-streptomycin. African green monkey kidney cells (Vero E6), kindly provided by J.L. Whitton, were maintained in DMEM supplemented with 10% FBS, 1% penicillin-streptomycin and 1% HEPES. M.J. Buchmeier (University of California, Irvine) kindly provided baby hamster kidney (BHK-21) cells which were cultured in DMEM containing 10% FBS, 1% penicillin-streptomycin and 1% Glutamax. Cells were grown in a humidified incubator at 37°C with 5% CO_2_.

Recombinant (r)LCMV based on the Armstrong 53b strain, including the wild type virus as well as the Z-KK10/77RR, Z-NoK and Z-PPPY/AAAA mutants, were generated as previously described [76, 110]. The rLCMV Armstrong 53b reverse genetics system was generously provided by Juan Carlos de la Torre while the non-recombinant Armstrong 53b strain of LCMV was kindly provided by J.L. Whitton. The attenuated strain of JUNV XJ, JUNV C#1, was generously provided by M.J. Buchmeier and R. Tesh (The University of Texas Medical Branch at Galveston) and is described in [111, 112]. Vero E6 cells were used to generate working stocks of these viruses as well as for measuring their titers via standard plaque assay.

### Plasmids and primers

The LCMV Pol I L segment plasmids containing the KK10/77RR, NoK and PPPY/AAAA mutations in the Z protein were synthesized by BioBasic, Inc (Markham, Ontario, Canada) using the WT plasmid as a template for cloning. Plasmids expressing the LASV Z WT, JUNV Z WT, LCMV Z WT, Y88F, Y88E and Y88A proteins with a C-terminal linker (amino acid sequence AAGGGG) and streptavidin binding peptide (SBP) affinity tag have been described previously [57, 76]. The HA-tagged ubiquitin plasmid was purchased from Addgene (18712). For ubiquitin mapping experiments, the LCMV Z WT plasmid was modified by mutating the single lysine residue in the SBP tag to arginine in order to prevent the possibility of a ubiquitin modification within the affinity tag. This plasmid, as well as the mutant versions NoK, PPPY/AAAA, K5R, K10R, K33R, K38R, K69R and K77R were made by Biobasic, Inc. The nucleotide sequence of the WT Z gene matches NCBI gene identifier number AY847351 while the translated amino acid sequence for the WT Z gene matches Protein Locus number AAX49343. Plasmids LCMV Z K5 only, K10 only, K33 only, K38 only, K69 only and K77 only were constructed by two rounds of PCR amplification using the LCMV Z WT and LCMV Z-NoK plasmids containing the modified lysine-free SBP tag as the DNA template and primers to introduce the lysine to arginine mutations at the specific sites. In the first round of the PCR the 5’ half and 3’ half of the gene were amplified in separate reactions in which the reverse primer for the 5’ half and the forward primer for the 3’ half covered residue 5, 10, 33, 38, 69 or 77 and were used to introduce a lysine to arginine or an arginine to lysine mutation. After purifying the PCR products, a second round of PCR was used to fuse the 5’ and 3’ halves. The AttB1/AttB2-flanked PCR products were shuttled into a Gateway donor vector using the Gateway BP Clonase II enzyme (11789100, Thermo Fisher Scientific) then into a modified pCAGGS destination vector that has been previously described [55, 113] using the LR Clonase II enzyme (11791100, Thermo Fisher Scientific). The double point mutant KK10/77RR was generated essentially as described above using the K10 only and K77 only plasmids as templates. The plasmid expressing WT LCMV Z with a C-terminal Strep tag II contained the same linker between Z and the tag as the SBP constructs (amino acid sequence AAGGGG) and was synthesized by GenScript (Piscataway, NJ). The sequence of all plasmids was confirmed by automated DNA sequencing at the Vermont Integrative Genomic Resource DNA Facility at the University of Vermont.

The following primers were used for cloning reactions (purchased from Integrated DNA Technologies, Inc.):

AttB1-EcoRV-Kozak-LCMV Z Forward (5’-acaagtttgtacaaaaaagCAGGCTgatatcGCCACCatgggtcaaggcaAgtccaga-3’), AttB1-EcoRV-Kozak-LCMV Z K5R Forward (5’-acaagtttgtacaaaaaagCAGGCTgatatcGCCACCatgggtcaaggca***G***gtccaga-3’), SBP-Stop-G-AttB2 Reverse (5’-ACCACTTTGTACAAGAAAGCTGGGTCTTACGGTTCACGCTGACCCTGCGGG-3’), LCMV Z-K33R Reverse (5’-TCTGCCAGCAAGATCTGCAGCTTAAAGGGCCAAG-3’), LCMV Z-K33R Forward (5’-CTTGGCCCTTTAAGCTGCAGATCTTGCTGGCAGA-3’), LCMV Z-K38 Reverse (5’-TCTTACCAAGCTGTCAAATTTCTGCCAGCAAGATLCMV-3’), LCMV Z-K38 Forward (5’-ATCTTGCTGGCAGAAATTTGACAGCTTGGTAAGA-3’), Z- K69 Forward (5’-GACAGGTGTCCTCTTTGTAAATATCCATTACCAACCAGA-3’), LCMV Z-K69 Reverse (5’-TCTGGTTGGTAATGGATATTTACAAAGAGGACACCTGTC-3’), LCMV Z-K69R Reverse (5’-TCTGGTTGGTAATGGATATCTACAAAGAGGACACCTGTC-3’), and LCMV Z-K69R Forward (5’- GACAGGTGTCCTCTTTGTAGATATCCATTACCAACCAGA-3’).

### Transfections and affinity purifications

For plasmid transfections, HEK293T cells were seeded at 5x10^5^ cells per well in 6-well plates. Cells were transfected 24 hours after seeding with 2 μg of different LCMV Z plasmids, 2 μg of HA-Ub plasmids, and 4 μg of 1 mg/mL Polyethylenimine (PEI) (23966; Polysciences, Inc., Warrington, PA) per well. 48 hours after transfection the cells were treated with 50 μM of MG-132 (474790; Millipore Sigma, Burlington, MA), a proteasome inhibitor to prevent the degradation of ubiquitin-containing proteins, for 2 hours before harvesting. For transfection experiments involving compound #4, cells were seeded and transfected with LCMV Z WT and ubiquitin plasmids as above. Then, the cells were treated with compound #4 24 hours after transfection. Compound #4 (Amb123203) was purchased from Ambinter (Orléans, France) and has been described previously in [88].

For transfection with both siRNA and plasmids, HEK293T cells were seeded at 2.5x10^5^ cells per well in 6-well plates that were treated with 100 μg/mL aqueous solution of poly D-lysine Hydrobromide (P6407; Sigma Aldrich, St. Louis, MO) then washed with PBS. 24 hours after seeding, 1.5 μL each of 10 μM siRNA targeting Nedd4, ITCH and WWP1 or 4.5 μL of a non-targeting control siRNA were added to a solution containing 7.5 μL of Lipofectamine RNAiMax (13778075; Life Technologies) in a final volume of 500 μL of Opti-MEM (31985070, Thermo Fisher Scientific) and incubated at room temperature for 20 minutes. 500 μL of this solution was added to the cells with an additional 2.5 mL of Opti-MEM. The media was replaced with fresh Opti-MEM 24 hours after siRNA transfection. Cells were transfected with LCMV Z and ubiquitin plasmids 48 hours after initial siRNA transfection using PEI as above and were harvested 48 hours after transfection with plasmids. The following siRNAs were purchased from Thermo Fisher Scientific: Silencer Select siRNAs (4390824, Life Technologies) targeting ITCH (assay ID s38163), NEDD4 (assay ID s9415), WWP1 (assay ID s21789) and Silencer Select control siRNA #1 (4390843).

Cells were harvested by scraping into media or PBS, pelleted by centrifugation, and then were lysed with 1x Triton Lysis Buffer (0.5% NP40, 1% Triton X-100, 140 mM NaCl, and 25 mM Tris-HCl containing a protease inhibitor cocktail (04693159001, Roche Applied Science, Indianapolis, IN)). SBP-tagged Z protein was affinity purified using magnetic streptavidin beads (65601, Thermo Fisher Scientific) and was eluted in Laemmli sample buffer containing 5% 2-mercaptoethanol by boiling at 100°C for 10 minutes. Affinity purifications done with strong denaturing conditions were performed essentially as above except urea powder (U5378, Sigma Aldrich) was added to the cell lysates to a final concentration of 8 M and was incubated for 1 hour at room temperature. The concentration of urea was decreased by diluting the solution with 1x Triton lysis buffer then the affinity purification with magnetic streptavidin beads was performed at this lower concentration of urea for 7 to 8 hours at 4°C.

### Virus challenge assays

For RNA interference virus challenge, 1 mL of A549 media containing 4x10^4^ A549 cells was combined with 2 μL of RNAiMax, 1.2 μL of 10 μM siRNA and 196.8 μL of Opti-MEM in a 12-well plate. In addition to the non-targeting siRNA and the siRNAs targeting ITCH, Nedd4 and WWP1 listed above, Silencer Select siRNAs (4390824, Thermo Fisher Scientific) targeting HGS (s17482) and TOM1 (s19514) were also used. The cells were infected 48 hours following reverse transfection with LCMV at a MOI of 0.001 plaque forming units (PFU) per cell. Recombinant (r)LCMV was used for challenges targeting the HGS and TOM1 proteins while non-recombinant LCMV was used for challenges targeting Nedd4 family proteins. The inoculum was replaced with 1 mL of fresh A549 media 1 hour after infection. 48 hours after infection the cells and virus-containing media were collected. Viral titers were determined via plaque assay and each titer value was first normalized to the sum of all titer values for a given independent experiment and the quotient was then divided by the mean quotient of the non-targeting siRNA for all independent experiments. All values were then multiplied by 100 to obtain the titer values as a percent of the control. This method of normalization by summation is described in [114]. For compound #4 and compound #5 challenges, 2.5x10^5^ HEK293T cells were seeded in 6-well plates then the next day were infected with non-recombinant LCMV at a MOI of 0.001. One hour after infection, the inoculum was removed at replaced with HEK293T media containing DMSO or the designated concentrations of compound #4 or #5. Two days later media was collected and titered by standard plaque assay and the titers were normalized as above. Compound #5 (Amb21795397) was purchased from Ambinter (Orléans, France) and has been described previously in [88].

### Plaque interfering assay

Titers of defective interfering particles were measured as previously described [76] and the assay is based on the plaque reduction assay described in [115]. For this assay, 2x10^4^ Vero E6 cells were seeded in each well of a 24-well plate in 0.4 mL of media. The next day, samples to be titered were thawed and transferred into clear snap cap tubes (21-402-904, Thermo Scientific) then a UVP CL-1000 ultraviolet crosslinker was used to irradiate the samples for two minutes with ultraviolet light to neutralize infectious virus. Each sample was subsequently serially diluted five times in five-fold increments then 50 μL per well of the undiluted or diluted samples were added to each row of the 24-well plate after approximately half of the media in each well was aspirated. A volume of 50 μL containing 50 PFU of non-irradiated rLCMV WT was then added to each well in three rows of the plate, but not to the fourth row (used as a reference row to ensure that each sample of interest had been completely irradiated). Plates were incubated for 60–90 minutes at 37°C then each well was overlaid with a 0.7% agarose (20–102, Apex Industrial Chemicals, Aberdeen, United Kingdom) in Vero growth media. After five days, the plates were fixed with formaldehyde and the monolayers were stained with 0.1% crystal violet (C581-100, Fisher Scientific) and 2.1% ethanol in water. The plaques were counted in each well containing the irradiated sample of interest and 50 PFU of standard virus. The mean number of plaques in each column of the three replicate rows was determined and the plaque reduction statistical web tool (https://bioinformatics.niaid.nih.gov/plaquereduction) was used to calculate the plaque interfering units 50 (PIU_50_). For this calculation, the mean number of plaques per well from an entire 24-well plate, in which only 50 PFU of standard virus was added to each well, was used as the control value required for this web tool.

### SDS-PAGE and western blotting

Cell lysates were prepared in Laemmli sample buffer such that the final concentration was 5% 2-mercaptoethanol and heated for 10 minutes at 70°C. Cell lysates and affinity purified samples were separated by protein gel electrophoresis using NuPAGE 4–12% Bis-Tris gels with MES buffer (catalog number NP0002; Invitrogen). Proteins were transferred to nitrocellulose membranes using iBlot 2 transfer stacks (IB23001 or IB23002, Thermo Fisher Scientific) and the iBlot2 western device as directed by the manufacturer. To confirm successful transfer of protein, the membranes were stained with a solution containing 0.1% Ponceau S (P3504, Sigma-Aldrich) and 5% acetic acid which was subsequently removed by washing with water. The membranes were probed using the iBind Flex western device (SLF2000, Thermo Fisher Scientific) and the antibodies were diluted in iBind Flex fluorescent detection solution (SLF2019, Thermo Fisher Scientific) or in iBind Flex solution (SLF2020, Thermo Fisher Scientific) following the manufacturer’s instructions. Nedd4, however, was probed for using traditional western blotting techniques according the antibody manufacturer’s instructions. The LI-COR Odyssey CLx imaging system was used for detection of fluorescent western blots while chemiluminescent blots were detected using a LI-COR C-Digit imaging system or a General Electric Amersham Imager 600. Quantitative analysis of western blots was performed using LI-COR Image Studio software.

The primary antibodies were used for western blotting (concentrations as indicated): mouse anti-streptavidin binding peptide (MAB10764, Millipore) (1:5,000), rabbit anti-streptavidin binding peptide (ab119491, Abcam, Cambridge, MA) (1:500), mouse anti-HA (16B12, Covance) (1:1,000), mouse anti-HGS (sc-271455, Santa Cruz Biotechnology, Dallas, TX) (1:250), mouse anti-TOM1 (sc-514430, Santa Cruz Biotechnology) (1:1,000), rabbit anti-ITCH (SAB 4200036, Sigma Aldrich) (1:5,000), mouse anti-WWP1/AIP5 (sc-100679, Santa Cruz Biotechnology) (1:500), rabbit anti-Nedd4 (2740, Cell Signaling), (1:1,000), rabbit anti-VPS4A/B (SAB 420025, Sigma-Aldrich) (1:1,000), mouse anti-ATP5B (sc-166443, Santa Cruz Biotechnology) (1:1,000), rabbit anti-IMPDH2 (ab 131158, Abcam) (1:1,000), rabbit anti-LCMV nucleoprotein (2165) (1:5,000), mouse anti-JUNV nucleoprotein clone NA05-AG12 (NR-2582; BEI Resources) (1:200), mouse anti-β-actin (A5441; Sigma-Aldrich) (1:5,000), rabbit anti-actin (A2066; Sigma-Aldrich) (1:5,000). The secondary antibodies were used for western blotting (concentrations as indicated): goat anti-rabbit IRDye 800CW (926–32211, LI-COR) (1:3,000), goat anti-mouse IRDye 680LT (926–68020, LI-COR) (1:4,000), goat anti-mouse IRDye 800CW (926–32110, LI-COR) (1:3,000), goat anti-mouse horseradish peroxidase (HRP) antibody (71045, EMD Millipore) (1:5,000), and goat anti-rabbit (HRP) (111-035-045, Jackson ImmunoResearch Laboratories) (1:5,000).

### Virus growth curve

To measure the growth of rLCMV harboring various mutations, 1.5x10^5^ A549 cells were added to each well of a 6-well plate. 24 hours after seeding the viruses were infected at a MOI of 0.01 PFU/cell and after 1 hour the inoculum was removed, the monolayers were washed once with 37°C PBS, then 2 mL of fresh A549 media was added to each well. At 1, 16, 24, 40, 48 and 72 hours after infection the media from one well was collected (a separate well was used for each virus at each time point) and clarified by centrifugation. Each sample was titered by standard plaque assay.

### Quantitative RT-PCR and sequencing of rLCMV

Extractions of viral RNA from cell-free virions was performed using the viral RNA mini kit (52904, Qiagen, Valencia, CA). For quantitative PCR, cDNA was generated for the LCMV S-segment using primer S 2865 (5’-CAGGGTGCAAGTGGTGTGGTAAGA-3’) and for the L-segment using primer L 5906- (5’-TGGGACTGAGTTTCGAGCATTACG-3’). Multiscribe reverse transcriptase (4308228, Applied Biosystems) was used in a 50 μL reaction to generate cDNA as described previously [76, 116]. Quantitative PCR was performed using 5 μL of cDNA product, 12.5 μL of Taqman universal PCR master mix (4326614, Thermo Fisher Scientific) and 2.5 μL each of the forward and reverse primers at 9 μM and the probe at 2 μM. The S segment was amplified with primers S 2275+ (5’-CGCTGGCCTGGGTGAAT-3’) and S 2338- (5’-ATGGGAAAACACAACAATTGATCTC-3’) and probe S 2295+ (5’-6FAM-CTGCAGGTTTCTCGC-MGBNFQ-3’). The L segment was amplified with primers L 5517+ (5’- GGCCTTGTATGGAGTAGCACCTT-3’) and L 5645- (5’- GGTCTGTGAGATATCAAGTGGTAGAATG-3’) and probe L 5582- (5’-6FAM-CTGAAGAATACCACCTATTATACCA-MGBNFQ-3’). An Applied Biosystems StepOnePlus real-time PCR system was used for quantitative PCR reactions. The copy numbers of each genome segment were calculated by standard curve analysis using plasmids that contain the full S or L genome segment as previously described [76, 116].

In order to sequence the rLCMV strains generated here, cDNA was generated using 5 μL of purified viral RNA, 1 μL of primers L 7219- (5’-GCACCGAGGATCCTAGGCTTTTTGATGCGCAA-3’) or S 3376- (5’-CGCACAGTGGATCCTAGGCATTTGA-3’) at 2 μM, 1 μL of 10 mM dNTPs, 6 μL nuclease-free water, 1 μL of Superscript III (18080–044, Thermo Fisher Scientific), 1 μL of RNAse Out (10777–019, Thermo Fisher Scientific), 4 μL of 5x first strand buffer and 1 μL of 0.1 M dithiothreitol according to the manufacturer’s protocol. PCR amplification of regions of the genome segments was conducted with Platinum SuperFi DNA polymerase (12351050, Thermo Fisher Scientific) following the manufacturer’s protocol. The following primer pairs (and the annealing temperature used for thermocycling) were used to amplify regions of the S and L segments: S 3357- (5’-ATTTGATTGCGCATTTGTCTTGAG-3’) and S 2031+ (5’-CAAGATCCATGCCGTGTGA-3’) (63°C), S 2628- (5’-GAACAGCAGTCCAGCATCAAC-3’) and S 861+ (5’-AGGAGACTAGCGGGCACATTC-3’) (64°C), S 1163- (5’-TCTCAAGTGGTTCCTCATCAGTAG-3’) and S 34+ (5’-TTTCCTCTAGATCAACTGGGTGTC-3’) (64°C), L 7227- (5’-GCACCGAGGATCCTAGGCTTTTTG-3’) and L 5321+ (5’-TTTGTTGGGTTGGTGAGCATTAGC-3’) (67°C), L 5816- (5’-TTGGAGCTCACCCGATAATG-3’) and L 3634+ (5’-GCCACACTGATCTTTAATGACTGA-3’) (63°C), L 4105- (5’-GCTGTGAGTCAAATTACGGAGAGT-3’) and L 1459+ (5’-GCTCTCAGGCTTTCTTGCTACAT-3’) (65°C), L 2155- (5’-TTGTAAGGAAACTGCATCATAAG-3’) and L 741+ (5’-TATTCTGATTGTGCGTAAAGTCCA-3’) (60°C), L 1461- (5’-GGGAGATGTTGTGTGTAATGCTGCCATGTTGA-3’) and L 2+ (5’-GCACCGGGGATCCTAGGCGTTTAGTT-3’) (two step cycling). The PCR products were digested with ExoSAP-IT (78200, Affymetrix, Santa Clara, CA) then sent for sequencing at the Vermont Integrative Genomic Resource DNA Facility at the University of Vermont.

### Identification of cellular protein partners of Z and LCMV virion content

To identify cellular proteins that interact with LCMV or LASV Z, two 6-well plates per condition were seeded with 5x10^5^ HEK293T cells per well and the next day each well was transfected with 2 μg of an empty vector or plasmids expressing SBP-tagged LCMV or LASV Z using PEI as described above. Two days after transfection, both the cells and VLPs were collected. The cell culture media containing VLPs was cleared of cellular debris by centrifugation then the cells and VLPs were lysed with triton lysis buffer containing protease inhibitor cocktail (04693159001; Roche, Indianapolis, IN) and PhosphoStop phosphatase inhibitor cocktail (4906837001; Roche). SBP-tagged Z was affinity purified with streptavidin beads as described above then protein complexes were separated by protein gel electrophoresis using a NuPAGE 4 to 12% Bis-Tris gradient gel. Following imaging of the Coomassie-stained gel with a Canon Canoscan 8800F scanner, each lane was cut into 16 slices. The gel samples were processed for and analyzed by LC-MS/MS as described in [57]. Briefly, samples were reduced with dithiothreitol, alkylated using iodoacetamide, then subjected to in-gel tryptic digestion. Peptides were extracted from the gel slices with acetonitrile and formic acid then were lyophilized in a vacuum centrifuge at 37°C. The samples were resuspended in 2.5% acetonitrile and 2.5% formic acid prior to separation by liquid chromatography and acquisition of mass spectra using a Thermo Scientific LTQ XL linear ion trap mass spectrometer. SEQUEST software was used to query the human IPI forward and reverse concatenated databases for which a 2 Da mass tolerance was permitted for tryptic peptides. Precursor mass differences of +79.96633 Da for serine, threonine, and tyrosine residues, +15.99492 Da for methionine residues, and +57.02146 or +71.0371 Da for cysteine residues were allowed for spectral analysis. Proteins which were identified with only one unique peptide were excluded from the dataset as were peptides with XCorr scores of less than 2.0. Requiring two peptides per protein under these filters led to a peptide false discovery rate of less than 1% for each dataset. Finally, proteins that were identified both in a Z condition and the empty vector control were eliminated unless there were five times more total peptides in the Z condition as in the empty vector.

To identify cellular proteins recruited into LCMV virions, Vero E6 cells were infected with the Armstrong 53b strain of LCMV and after two days, sucrose banding was used to purify cell-free virions as described previously [76, 117]. The purified virions were lysed with Triton buffer, prepared in Laemmli buffer with a final concentration of 5% 2-mercaptoethanol then the proteins were separated by SDS-PAGE. The gel slices containing the viral protein were subject to in-gel reduction, alkylation and tryptic digestion as was done for the Z protein samples above. The tryptic peptides were further processed and subjected to LC-MS/MS as described above.

### VLP release assays

For VLP release assays in Figs 8F, 9J and 9K, 2x10^5^ HEK293T cells in 0.8 mL of growth media per well were seeded in a 12-well plate then 24 hours later, the cells were transfected with 0.8 μg of Z plasmid and 3.2 μg of 1 mg/mL PEI per well. For the VLP release assays treated with compound #4 (Fig 9J and 9K), at the time of transfection, the media was replaced with fresh media containing either DMSO or 5 μM of compound #4, then the transfection mix was added. For the VLP release assay with siRNA knockdown of select targets (Fig 9D and 9E), 12-well plates were first treated with a 100 μg/mL aqueous solution of poly D-lysine hydrobromide then washed with PBS. 1x10^5^ HEK293T cells, siRNA at a 10 nM final concentration, and 1.2 μL of RNAiMax were combined in antibiotic-free HEK293T growth media in a final volume of 1.2 mL per well. The siRNAs used in this assay were the same as listed above except the siRNA targeting ATP5B (assay ID s1773). 24 hours after reverse transfection, the media from each well was replaced with 0.8 mL of antibiotic-free HEK293T growth media. 48 hours after siRNA reverse transfection, the cells were transfected with Z plasmids and PEI as above. For all VLP assays, 24 hours after plasmid transfection Triton lysis buffer was used to lyse the cells and clarified VLP-containing media and the Z protein was detected by quantitative western blot analysis. To calculate the percent VLP release, the quantity of each Z protein band was first divided by the sum of the quantities of all bands on each blot (normalization by summation as described in [114]). This normalized quantity of Z for VLPs was divided by the quantity of normalized intracellular Z and the quotient for each sample was divided by the mean of the control values and set to 100 percent.

### Bioinformatic and statistical analyses

In order to ensure that the gene or protein identifiers used were current and consistent across datasets for bioinformatics analyses, the DAVID Gene ID conversion tool [77, 78] was used to verify and convert gene symbols derived from the human IPI database, ScanProsite and literature sources. Identifiers not recognized by DAVID were manually searched for using NCBI Genbank [118] or GeneCards [119]. The DAVID gene functional classification tool (version 6.8) was used to identify proteins that were enriched in the Z interactome. The ScanProsite tool was used to search for P(S/T)AP, YPX_(1,3)_L and PPXY motifs in human proteins (taxon 9606) listed in the UniProtKB database [80, 81]. Human WW domains and ESCRT proteins were identified using references [82] and [14]. Overrepresentation of ESCRT proteins or proteins containing late domains or WW domains was determined by partitioning the set of human proteins according to whether they contained a given domain or were part of the ESCRT complex and whether they interacted with intracellular Z, VLP-derived Z or were found in LCMV virions. The contingency tables were assessed for significance using the Fisher Exact Test implemented in R to determine odds ratios, confidence intervals, and p-values [120]. The Benjamini-Hochberg FDR method was used for multiple comparison p-value adjustment [121]. All other statistical analyses were performed using GraphPad Prism software version 7.01.

## Supporting information

S1 Table Human proteins that interact with LCMV Z or LASV Z protein in Z-transfected cells or in VLPs released from Z-transfected cells. Human proteins recruited into *bona fide* LCMV Armstrong 53b virions are also shown. The Official Gene Symbol, Entrez ID, Gene Description, and IPI ID are listed for each identified protein. The spectral counts (total peptides) for each condition and experimental replicate are listed for each identified protein. A heat map based on spectral counts is overlaid where green represents the lowest values, yellow the median and red the highest values. Whether each identified protein contains a PPXY, P(S/T)AP or YPX(1,3)L late domain, WW domain, or is part of the ESCRT pathway is also indicated.
(XLSX)

S2 Table Human proteins enriched in proteomics datasets. Cellular proteins identified in cells or VLPs released from LCMV or LASV Z-transfected cells or in LCMV virions were analyzed using the NIH DAVID (version 6.8) gene functional classification tool using the high stringency setting and with Homo sapiens as the background. Bolded genes were identified in both replicate experiments.
(XLSX)

S3 Table Bioinformatic identification of specific human protein classes. ScanProsite was used to identify human cellular proteins that contain a PPXY, P(S/T)AP or YPX(1,3)L late domain, WW domain, or that are part of the ESCRT pathway.
(XLSX)
